# Architecture of the brain’s visual system enhances network stability and performance through layers, delays, and feedback

**DOI:** 10.1371/journal.pcbi.1011078

**Published:** 2023-11-10

**Authors:** Osvaldo Matias Velarde, Hernán A. Makse, Lucas C. Parra

**Affiliations:** 1 Biomedical Engineering Department, The City College of New York, New York, New York, United States of America; 2 Levich Institute and Physics Department, The City College of New York, New York, New York, United States of America; Brown University, UNITED STATES

## Abstract

In the visual system of primates, image information propagates across successive cortical areas, and there is also local feedback within an area and long-range feedback across areas. Recent findings suggest that the resulting temporal dynamics of neural activity are crucial in several vision tasks. In contrast, artificial neural network models of vision are typically feedforward and do not capitalize on the benefits of temporal dynamics, partly due to concerns about stability and computational costs.

In this study, we focus on recurrent networks with feedback connections for visual tasks with static input corresponding to a single fixation. We demonstrate mathematically that a network’s dynamics can be stabilized by four key features of biological networks: layer-ordered structure, temporal delays between layers, longer distance feedback across layers, and nonlinear neuronal responses. Conversely, when feedback has a fixed distance, one can omit delays in feedforward connections to achieve more efficient artificial implementations.

We also evaluated the effect of feedback connections on object detection and classification performance using standard benchmarks, specifically the COCO and CIFAR10 datasets. Our findings indicate that feedback connections improved the detection of small objects, and classification performance became more robust to noise. We found that performance increased with the temporal dynamics, not unlike what is observed in core vision of primates.

These results suggest that delays and layered organization are crucial features for stability and performance in both biological and artificial recurrent neural networks.

## Author summary

The visual cortex is a part of the brain that receives, integrates, and processes visual information. It is made up of many interconnected areas that work together to help us see. Studies have shown that lateral and feedback connections between these areas are essential for us to be able to see and understand the world around us. However, most computer vision models only consider feedforward connections.

In this study, we looked at the stability of networks with feedback. We used mathematical tools to discover that layered networks with long-range feedback favor stability, as do biologically realistic implementations with temporal delays in the feedforward connections. We also demonstrated the performance advantages of adding feedback connections to convolutional networks in image classification and detection tasks.

These results suggest that the organization of the visual system favors stability. This implies that biologically more realistic implementations of computational vision networks may be easier to train.

## 1 Introduction

**Fig 1:**
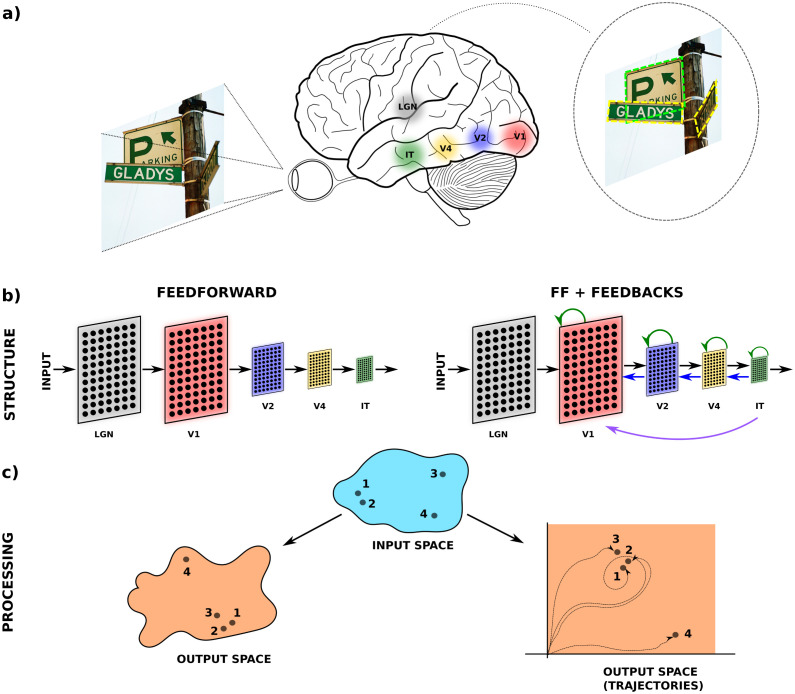
Visual processing. (a) Schematic of visual processing across several areas defined by anatomical structure and function of the area such as V1, V2, V4, and Inferior Temporal cortex (IT). The major input to the visual cortex is from the lateral geniculate nucleus (LGN) whose principal neurons receive input from the retina [26]. (b) Models of the visual cortex. In feedforward models, information is only processed in one direction. In models with feedback connections, information from distant layers loops back to earlier layers (e.g. from IT to V1) across multiple distances [27]. Also, local activity feeds back to the same layer. (c) Feedforward models map each point in the input space to a point in the output space. In a model with feedback, a static point in the input space can generate a temporal dynamic (or trajectory) in the output space. The properties of the trajectory strongly depend on the feedback connections and the input. This image is inspired by Fig 2 in [28] describing “core vision”, the sub-second processing in primates during a single fixation.

The visual system receives information arriving from the retinas through the lateral geniculate nucleus and processes it from there through a sequence of cortical areas (see Fig 1a) [1, 2]. Each subsequent cortical area captures a hierarchy of image information transforming low-level visual features, such as edges in V1, to mid-level shapes in V2-V4 to high-level semantic in IT (Inferior Temporal cortex) [1, 3–5]. This processing takes a few hundred milliseconds and typically happens with a nearly-static input while the animal maintains fixation [3].

Computer vision has taken inspiration from this hierarchical organization to design image-processing networks that solve a variety of vision tasks [6–8]. These artificial neural networks are organized in successive layers often with identical processing within a layer that can be implemented as a set of convolutions (Fig 1b). The term “layer” in neuroscience refers to distinct layers of neuronal structures on the cortical sheet within a single cortical area, whereas in computational networks it refers to a sequence of similar processing stages comparable to the sequence of processing across cortical areas. Here we will use the meaning of layer used in computational networks. Such convolutional neural networks (CNN) have achieved remarkable performance when the network is many layers deep. In such deep networks, “activations” at various depths have been associated with neural activity observed in different areas of the visual processing hierarchy [9, 10]. An early finding was that neural activity in IT contains enough information to identify the class of the image comparable to deeper layers in CNNs [3, 11].

Conventional CNNs consist of convolutions followed by nonlinear functions applied sequentially without feedback connections (Fig 1b-left). However, both anatomical and functional evidence in the primate visual system show the presence of a large number of lateral connections within brain areas, and feedback connections from later to earlier areas [12, 13]. It is now well established that these distant feedback connections contribute to visual processing [14–18] (Fig 1b-right). Importantly, feedback connections lead to a *temporal dynamic* as the activity in a given area will change once it receives feedback from later (higher) areas. Thus, even in the presence of a static input, the neural activity follows *trajectories* (Fig 1c-right) in the space of activations.

The structure of the network determines the temporal dynamic including the time-scale of changes in activations [19], the location of fixed points or limit cycles and their stability [20]. Feedforward networks are by definition stable. In contrast, a network with feedback is not guaranteed to have stable temporal dynamics and the question of stability becomes important. The most dramatic example of instability in a biological network is runaway excitation during epileptic seizures. For biological neural networks to be effective, it is critical that their dynamics reach a consistent state (or sequence of states) [21, 22]. Meanwhile, for artificial neural networks [23], stability plays an important role in the learning process [24, 25].

In visual processing, the dynamics of the network define how the input (image) will be processed to obtain the output (representations) (Fig 1c). For feedforward processing, each point in the input space is associated with a point in the output space. For a network with feedback connections, however, each input image results in a trajectory of activations, even when the input is constant. In visual tasks where only high-level representations matter, the output should be robust to perturbations of the input, such as partial occlusion, orientation, and contrast. For example, in Fig 1c, assume that points 2 and 3 of the input space represent different perturbations of input point 1, and their corresponding outputs are close. Note that point 3 is a stronger disturbance than point 2 (comparable to the distance of an arbitrary point 4); however, the input-output relationship correctly replicates the desired proximity relations.

In this work, we use analytic tools of dynamical system theory to study the stability of networks with local and long-range feedback connections. We found that the stability of the network depends on the temporal delay of the feedforward connections. We also show that feedback over longer distances and a layered structure favor the stability of the dynamics. Finally, we use these results to add stable feedback in recurrent convolutional networks for visual tasks such as object detection and classification. There, feedback connections improved the detection of small objects and robustness against noise.

## 2 Methods

A neural network consists of connected units, with connections characterized by their sign, strength, and time delay. The structure of connections determines the resulting dynamics of activity in the units of the network. When connections are organized in a sequence of layers, it’s possible to distinguish between feedforward and feedback connections (a mathematical definition is provided in Section A.1 of S1 Text). To analyze the stability of the dynamic we will consider a simplified network, first with single units per layer, and later extend this to multiple units per layer. To analyze the effects of feedback on performance in visual tasks we then rely on a state-of-the-art convolutional network with a more complex structure. Here we introduce both structures.

### 2.1 Reduced neural network

In an artificial neural network that has only feedforward connections, there is no need to consider time delays, and connections are often treated as instantaneous. Once feedback is included, one has to decide on the exact order of operations, and whether the forward pass is implemented instantaneously, or if each transmission from one unit to the next takes time, as it does in a biological system. To make these notions concrete, consider the following dynamics of the variable $h_{l,t}\in R$ that represents the activation in layer *l* ∈ {1, 2…, *N*} (with a single unit) at time *t*:

$$\begin{array}{c} h_{l,t}=x\delta_{l,1}+\alpha_{l-1\to l}h_{l-1,t-\Delta }+\sum_{k=l}^{N}\;\alpha_{k\to l}h_{k,t-1}. \end{array}$$ (1)

where x∈R is a constant input in the first layer (*l* = 1), *α*_*j*→*i*_ and Δ indicate the weight and time delay of the connection between layers *j* → *i*, respectively. We use the symbol *δ* to represent the Kronecker delta.

**Fig 2:**
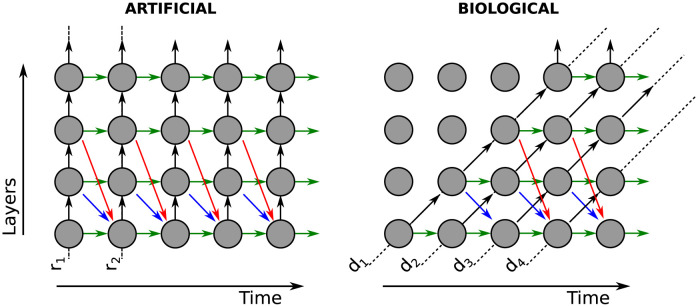
Two implementations of feedforwad transmission. In the artificial case, feedforward connections (black arrows) transmit information instantaneously (Δ = 0). In the biological case, feedforward transmission requires time and thus introduces a time delay (Δ = 1). In both cases, feedback connections require a delay (Δ = 1) irrespective of distance (0, 1, or 2 in green, blue, and red). Note that in the artificial case, the units along lines *r*_*i*_ only depend on the information from units along line *r*_*i*−1_; while in the biological case units along line *d*_*i*_ depend on the information from units along several lines *d*_*i*−1_, *d*_*i*−2_, …. The lines *r*_*i*_ and *d*_*i*_ represent how the state of the network evolves as a function of the layers and time.

When feedforward connections are instantaneous (Δ = 0), we say that the transmission of information is *Artificial*; while for Δ = 1 we say that it is *Biological* (Fig 2).

The matrix version of the Eq (1) is:

$$\begin{array}{c} {\vec{h}}_{t}=x{\vec{e}}_{1}+M_{FF}{\vec{h}}_{t-\Delta }+M_{FB}{\vec{h}}_{t-1} \end{array}$$ (2)

where ${\vec{h}}_{t}=\left(h_{1,t},h_{2,t},\ldots ,h_{N,t}\right)\in R^{N}$, and ${\vec{e}}_{1}=\left(1,0,\ldots ,0\right)\in R^{N}$. The *N* × *N* matrices are defined by (*M*_*FF*_)_*ij*_ = *α*_*j*→*i*_ *δ*_*j*,*i*−1_, and (*M*_*FB*_)_*ij*_ = *α*_*j* → *i*_ Θ(*i* ≤ *j*). Here, Θ indicates the step function.

Regardless of the value of Δ, the fixed point of the system is

$$\begin{array}{c} {\vec{h}}^{*}=x{\left(Id-M_{FF}-M_{FB}\right)}^{-1}{\vec{e}}_{1} \end{array}$$ (3)

However, the stability of ${\vec{h}}^{*}$ depend on Δ. Let’s rewrite Eq (2) for the two cases:

- Biological: (Δ = 1): ${\vec{h}}_{t}=x\vec{e}+M{\vec{h}}_{t-1}$ where *M* = *M*_*FF*_ + *M*_*FB*_
- Artificial: (Δ = 0): ${\vec{h}}_{t}=x{\left(Id-M_{FF}\right)}^{-1}\vec{e}+M{\vec{h}}_{t-1}$ where *M* = (*Id* − *M*_*FF*_)^−1^*M*_*FB*_

Both of the above equations define a discrete-time linear dynamical system. The fixed point h* and its stability depend on the entries of the matrix *M*. From the bifurcation theory, we know that the eigenvalues of the matrix *M* define the stability of the system [29]. By definition, λ is an eigenvalue of the matrix *M* if and only if it is a root of the characteristic polynomial *p*_*M*_(λ) = *det*(*M* − *λId*). When all the eigenvalues satisfy |λ| < 1, the iteration above will converge to a fixed point. However, when there is at least one eigenvalue with |λ| > 1, the iteration will diverge. The bifurcation boundary between stable and unstable dynamic can thus be parameterized by the equation *p*_*M*_(*e*^*iθ*^) = 0, since |*e*^*iθ*^| = 1 with *θ* ∈ [0, 2*π*).

Note that a layer right now only has a single unit. Later we will treat the case where each layer contains several units. From now on, we will use the notation *α*_*j*,*i*_ = *α*_*j*→*i*_.

### 2.2 A recurrent convolutional neural network

**Fig 3:**
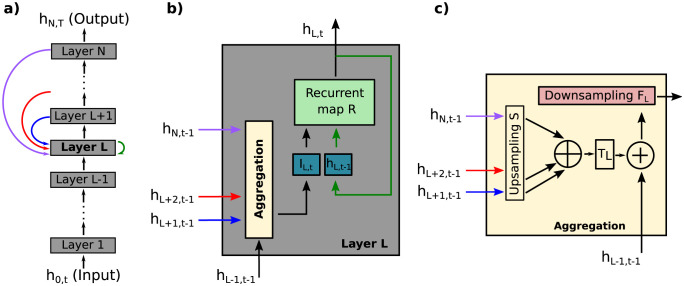
A recurrent CNN. (a) The CNN is composed of *N* layers (gray boxes) and bottom-up and top-down connections (black and colored arrows). Each bottom-up connection involves spatial downsampling or local pooling. The network input is a static image (i.e. *h*_0,*t*_ = *x*) and the output is the activity of the last layer after *T* time steps (i.e. *h*_*N*,*T*_). (b) Each layer *l* is composed of a mechanism for aggregating information from other layers (i.e. *h*_*l*−1,*t*_, *h*_*l*+1,*t*−1_, …, *h*_*N*,*t*−1_) and a recurrent map *R* that updates the activity of the layer *h*_*l*,*t*_—see Eq (4)—and transmits this to other layers. (c) The aggregation consists of upsampling feedback from higher layers with the function *S* to match spatial dimensions of the current layer and concatenating along the channel axis (⊕). Then a linear map *T*_*l*_ combines these channels resulting in the same number of channels as *h*_*l*−1,*t*_, to which this feedback input is then added—see Eq (6). Finally, *F*_*l*_ is an additional nonlinear mapping that implements downsampling.

For the effective processing of images, we will need a more complex network structure. We will rely on a specific recurrent CNN based on [30]. This network has *N* layers with the activity of the layer *l* ∈ {1, 2, …, *N*} at time *t* ∈ {1, 2, …, *T*} given by the recurrent map *R*:

$$\begin{array}{c} h_{l,t}=R\left(h_{l,t-1},I_{l,t}\right), \end{array}$$ (4)

where *I*_*l*,*t*_ is the input to this layer originating in other layers (Fig 3). Each layer has units arranged as an image array of height *H*_*l*_ and width *W*_*l*_, with separate units for *C*_*l*_ “channels”, so that the activity of the layer is $h_{l,t}\in R^{H_{l}\times W_{l}\times C_{l}}$. The temporal dynamic is initialized with *h*_*l*,0_ = 0, while the external input at the lowest layer of the network is kept constant, *h*_0,*t*_ = *x*.

There are several ways to define the map *R*. Here, we analyze the *Time Decay* dynamic governed by the equation

$$\begin{array}{c} h_{l,t}=\tau_{l}h_{l,t-1}+I_{l,t} \end{array}$$

where *τ*_*l*_ is the time scale of the layer *l*.

For any function *R*, the variable *I*_*l*,*t*_ represents the information coming from other layers, i.e.

$$\begin{array}{c} I_{l,t}=\phi_{l}\left(h_{l-1,t-1},h_{l+1,t-1},\ldots ,h_{N,t-1}\right). \end{array}$$ (5)

There are several ways to define the information integration mechanism (i.e. the function *ϕ*_*l*_). An example is shown in Fig 3c, which is represented by the equation:

$$\begin{array}{c} I_{l,t}=F_{l}(h_{l-1,t}+T_{l}(\overset{N}{\underset{k=l+1}{⊕}}S\left(h_{k,t-1}\right))) \end{array}$$ (6)

where *F*_*l*_ is a “ResNet stage” which involves downsampling (see Section A.6 of S1 Text), *S* is an upsampling operation to match spatial dimensions of activity from later layers *h*_*k*,*t*_, ⊕ indicates concatenation along the channel axis, and *T*_*l*_ is a linear map combining the concatenated channels and reducing the channel dimension to match *h*_*l*−1,*t*_ (see Fig 3c).

Note that Eq (1) is a simplified case of the model proposed in this section. In particular, it is the result of reducing the number of units and channels per layer to one and setting *F* as a linear function. Note that the CNN presented here used a time delay for the feedforward connections between layers (Δ = 1)—see Eq (5).

### 2.3 Network training

**Fig 4:**
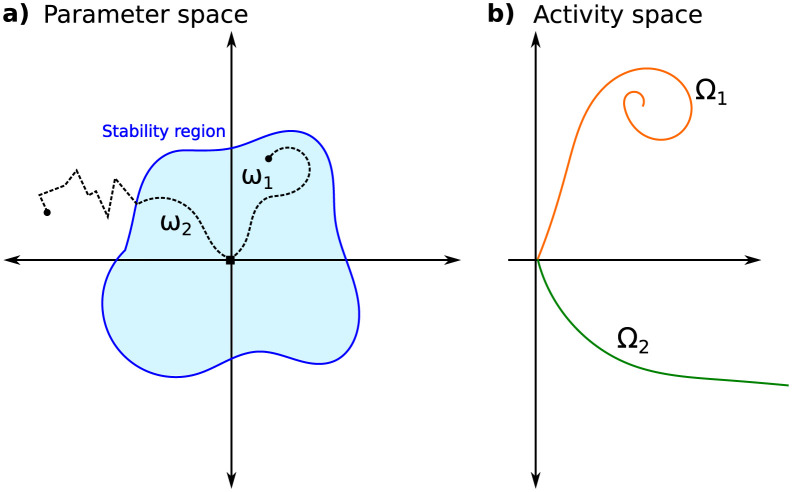
Training and inference dynamic. (a) Learning trajectories in parameter space. There exists a region in parameter space such that the dynamics of the activity are stable for those parameter values (inside the bifurcation boundary—blue curve). The trajectories *ω*_1_, *ω*_2_ start with stable dynamic. After some gradient updates the trajectory may remain in the stable domain (*ω*_1_) or may move beyond the stable domain (*ω*_2_). (b) Space of activity showing activity dynamic that converges (Ω_1_: orange curve corresponding to the endpoint of learning *ω*_1_) and dynamic that diverges (Ω_2_: green curve, corresponding to the endpoint of learning *ω*_2_).

To train these deep networks we use conventional gradient descent. In the training of recurrent neural networks, two levels of dynamics coexist:

1. The dynamics of network activity during inference: ${\vec{h}}_{t}=R\left({\vec{h}}_{t-1},x;w\right)$ with *t* = 1, …, *T*.
2. The dynamics of parameters during training: $w_{m}=w_{m-1}+r\nabla_{w}L\left(y,{\hat{y}}_{m-1}\right)$

where $L=L(y,\hat{y})$ is the cost function comparing the training labels *y* to the network prediction $\hat{y}$ for the input *x* (i.e. $x\mapsto {\vec{h}}_{T}\mapsto \hat{y}$). The activity dynamics during inference (1) take place in the space of activities ${\vec{h}}_{t}$, while the training dynamics (2) occur in the parameter space *w*. Computing gradients in (2) requires bounded activity dynamics (1) during inference. From an analytical perspective, the smoothness condition of the function is sufficient for gradient calculations; however, for computational purposes, it is necessary for the function and its derivative to be finite. The schematic of Fig 4a shows a training dynamic *ω*_1_ that remains for the entire duration in the domain of stable activation dynamic, exemplified by activity dynamic Ω_1_ in Fig 4b. In contrast, learning trajectory *ω*_2_ approaches the boundary of stability in the activity space, at which point learning can no longer progress smoothly as the activity diverges, exemplified by Ω_2_ in Fig 4b. When the dynamics of the activity converge to stable fixed points, it can be demonstrated that the fixed point’s dependence on the parameters is smooth (see Section 10.2 of [29]). This ensures the smoothness of $\nabla_{w}L$, thereby guaranteeing that trajectory *w*_*m*_ is, at the very least, continuous. On the other hand, bifurcations within the activity dynamics can disrupt the smoothness of the gradient and the continuity of trajectories within the parameter space.

One of the main theoretical results will be that the biological implementation of delays promotes stability. Indeed, initial simulation with artificial time delays often led to unbounded activity, making learning impossible in practice. Thus, in all scenarios presented here, networks with biological time delays were used. The training was initialized with all feedback weights set to zero ensuring that training starts with stable activity dynamics. We kept the conventional ReLU nonlinearity of these networks unchanged, which supports stability as it sets the gain to zero for all negative inputs (see Section 3.6). Additionally, we have retained batch-normalization used in these networks and included it for the feedback connections thus potentially further contributing to stability (Section 3.6). Beyond that, no additional measures were necessary to keep the networks in the space of stable activation dynamic, e.g. adding an explicitly “contractive” term as in [31] to promote stability during inference was not needed.

Finally, note that the notion of convergence of the training process (2) is not practically relevant for modern deep networks, where we typically use early stopping to prevent over-training. All networks we have trained here were not trained to convergence, and parameters stayed within the stability region without any additional constrained on learning.

## 3 Results

We will now demonstrate mathematically—using the reduced model of Section 2.1—that recurrent networks with biologically realistic delays are more stable than artificial networks with no delays in the forward connections (3.1). We also show that longer distance feedback increases stability (3.3), including when they are added to a network with shorter feedback connections (3.5). For the special case that a network only has feedback with a fixed distance, one can gain computational efficiency with an equivalent artificial implementation, without affecting stability (3.2). These basic results on stability still hold when the reduced model is extended to include layers with multiple units (Section A.2 of S1 Text). Indeed, when networks are organized in layers, as they are in biological networks, they gain stability as the relative strength of longer feedback tends to increase 3.4. The results presented next are derived for linear networks. However, we also show that for networks using typical nonlinear activation functions stability can only improve over the linear case when there are fixed points (3.6).

In Sections 3.7 and 3.8, we report on the performance benefits obtained for visual tasks when biological feedback is added to the recurrent CNN of Section 2.2.

### 3.1 A biological implementation with feedforward delay is more stable

As mentioned above, the bifurcation boundary is determined by the eigenvalues with absolute value equal to 1 (i.e. λ = *e*^*iθ*^, *θ* ∈ [0, 2*π*)). As we show in Section A.3 of S1 Text, λ = *e*^*iθ*^ is a root of $p\left(\lambda \right)=\sum_{n=0}^{N}c_{n}\lambda^{n}$ if and only if:

$$\begin{array}{c} \left\{ \begin{array}{cc} c_{0} & =\sum_{n=0}^{N-2}c_{n+2}U_{n}\left(z\right)\;\text{with}\;z=\text{cos}\;\left(\theta \right),\theta \neq 0,\pi \\ 0 & =\sum_{n=0}^{N-1}c_{n+1}U_{n}\left(z\right) \end{array}\right. \end{array}$$ (7)

or

$$\begin{array}{c} 0=\sum_{n=0}^{N}c_{n}{\left(-1\right)}^{mn}\;\text{with}\;\theta =m\pi . \end{array}$$ (8)

In the above equations, *U*_*n*_’s are Chebyshev polynomials of the second kind: *U*_0_(*z*) = 1, *U*_1_(*z*) = 2*z*, *U*_2_(*z*) = 4*z*^2^ − 1, … [32]. Note that the coefficients *c*_*n*_ of *p* depend on the values of the associated matrix (see examples in Table 1). We are interested in comparing the results for the matrices *M*_*B*_ = *M*_*FF*_ + *M*_*FB*_ and *M*_*A*_ = (*Id* − *M*_*FF*_)^−1^*M*_*FB*_ reflecting biologically realistic and artificial feedforward connections (see Section 2.1):

**Table 1:** Coefficients of characteristic polynomials. For matrices of size *N*, the coefficients of the characteristic polynomials are indicated as a function of the values of the matrix. For each size, the results are shown for the cases of biological and artificial feedforward connections. We denote *κ*_1_ = *α*_11_ + *α*_22_ + *α*_33_, *κ*_2_ = *α*_12_*α*_21_ + *α*_23_*α*_32_, *κ*_3_ = *α*_12_*α*_23_*α*_31_, *κ*_4_ = *α*_11_*α*_22_ + *α*_11_*α*_33_ + *α*_22_*α*_33_, *κ*_5_ = *α*_11_*α*_23_*α*_32_ + *α*_33_*α*_12_*α*_21_, *κ*_6_ = *α*_11_*α*_22_*α*_33_.

N	Coeff	Artificial	Biological
2	*c*_0_	*α*_11_ *α*_22_	*α*_11_*α*_22_ − *α*_12_*α*_21_
	*c*_1_	−(*α*_11_ + *α*_22_ + *α*_12_*α*_21_)	−(*α*_11_ + *α*_22_)
3	*c*_0_	-*κ*_6_	-(*κ*_6_ + *κ*_3_ − *κ*_5_)
	*c*_1_	*κ*_4_ + *κ*_5_	*κ*_4_ − *κ*_2_
	*c*_2_	−(*κ*_1_ + *κ*_2_ + *κ*_3_)	−*κ*_1_

For example, Eqs (7) and (8) for *N* = 2 are:

$$\begin{array}{cc} & \left\{ \begin{array}{c} c_{0}=U_{0}\left(z\right)=1\\ 0=c_{1}U_{0}\left(z\right)+U_{1}\left(z\right)=c_{1}+2\;\text{cos}\;\left(\theta \right)\;\text{with}\;\theta \neq 0,\pi \end{array}\right.\\ & 0=c_{0}+c_{1}+1,\;\theta =0\\ & 0=c_{0}-c_{1}+1,\;\theta =\pi \end{array}$$

And for *N* = 3 they are:

$$\begin{array}{cc} & \left\{ \begin{array}{cc} c_{0} & =c_{2}U_{0}\left(z\right)+U_{1}\left(z\right)=c_{2}+2\;\text{cos}\;\left(\theta \right)\;\text{with}\;\theta \neq 0,\pi \\ 0 & =c_{1}U_{0}\left(z\right)+c_{2}U_{1}\left(z\right)+U_{2}\left(z\right)=c_{1}+2c_{2}\;\text{cos}\;\left(\theta \right)+\left[4\;{\text{cos}}^{2}\;\left(\theta \right)-1\right] \end{array}\right.\\ & 0=c_{0}+c_{1}+c_{2}+1,\;\theta =0\\ & 0=c_{0}-c_{1}+c_{2}-1,\;\theta =\pi \end{array}$$

**Fig 5:**
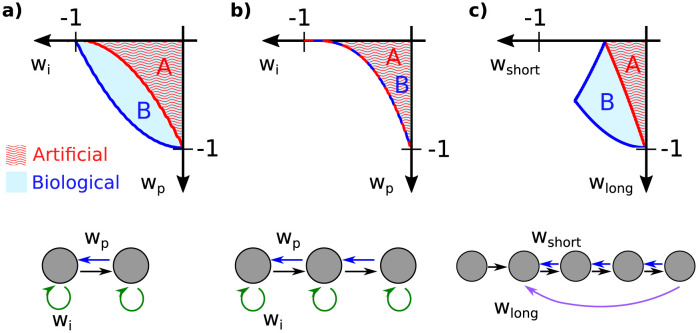
Bifurcation boundaries for 2, 3, and 5 layers. The region of the parameter space where the network is stable depends on the number of layers *N*, the type of feedforward transmission (B: biological with delay, A: artificial without delay), and the distance of the feedback (see Eqs (7) and (8)). For the networks presented in the lower panels (a) *N* = 2, (b) *N* = 3, (c) *N* = 5, we see that the stability region for the biological case is larger or equal than for the artificial case. This is a repeating behavior for different *N*. Notation: (a) *w*_*i*_ = *α*_11_ = *α*_22_, *w*_*p*_ = *α*_12_*α*_21_. (b) *w*_*i*_ = *α*_11_ = *α*_22_ = *α*_33_, *w*_*p*_ = *α*_12_*α*_21_ = *α*_23_*α*_32_ and *α*_31_ = 0. (c) *w*_*short*_ = *α*_23_*α*_32_ = *α*_34_*α*_43_ = *α*_45_*α*_54_, *w*_*long*_ = *α*_23_*α*_34_*α*_45_*α*_52_. Note that in all cases, the red and blue curves intersect at the axes (*w*_*i*_ = 0, *w*_*p*_ = 0, *w*_*short*_ = 0 or *w*_*long*_ = 0). This is a consequence of the discussion presented in Section 3.2.

In Fig 5, the region of the parameter space with stable dynamic for a few different network structures are shown. The structure in Fig 5c, in particular, is motivated by the simplified circuit diagram proposed for the ventral visual pathway, as shown schematically in Fig 1b, right. The regions of stability were determined analytically using Eqs (7) and (8) and the calculation of the coefficients *c*_*n*_ as functions of the weights *α*_*j*,*i*_ (e.g. Table 1). The results are shown for a 2D subspace of the parameter space for better visualization. However, in all cases, the following is true: *the stability region for networks with the biological transmission is greater than or equal to the stability region for the artificial case*. In the next section, we will identify the special cases where the stability regions of biological and artificial transmission are equal.

### 3.2 Cases where biological and artificial transmission are equivalent

We define *feedback of distance q* as connections from layer *l* to layer *l* − *q*, therefore *q* ∈ {0, 1, 2, …, *N* − 1}. In Fig 2, the green, blue, and red arrows are *feedbacks of distance* 0, 1, and 2, respectively.

For a fixed distance *q*, the weights of the connections *α*_*l*,*l* − *q*_ form one of the diagonals in the matrix *M*_*FB*_. For example, the weights *α*_11_, …, *α*_*NN*_ (i.e. *q* = 0) are on the main diagonal. On the other hand, *α*_21_, …, *α*_*N*,*N*−1_ (i.e. *q* = 1) are on the first off-diagonal.

**Fig 6:**
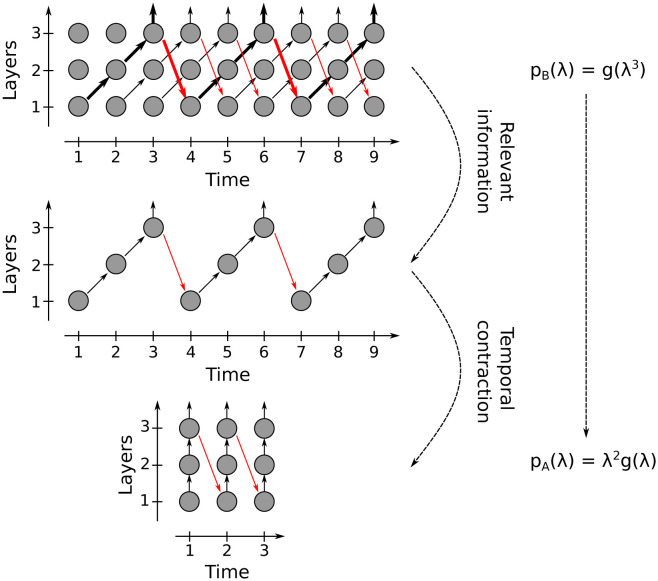
Equivalence between biological and artificial implementation. When the network consists of feedforward connections and single-distance feedback connections, the biological and artificial implementations have the same bifurcation boundaries. For a network with feedback connections of distance *q* = 2, the biological implementation (top panel) is represented with a pattern of arrows (bold lines) that repeats (q+1)-times. The complete information about the dynamics is in the pattern (center panel). When applying a temporal contraction, the pattern is equivalent to the information flow of the artificial implementation (bottom panel). The presented theorem (see main text) shows this equivalence through the transformation of the characteristic polynomial *p*_*B*_ to *p*_*A*_.

Suppose that in a network there are only feedforward connections (black arrows in Fig 2) and feedback connections of a fixed distance *q*. For example, let’s take *q* = 1 (blue arrows in Fig 2). Note that the black arrows form straight lines. In the artificial case, we denote them *r*_1_, *r*_2_, … and they are vertical lines; whereas in the biological case, we denote them *d*_1_, *d*_2_, … and they are diagonals. These lines are parallel to each other and only interact if there are feedback connections (blue arrows). The ordering of all the arrows indicates how information is transmitted across layers and over time. In the biological case, the blue arrows transmit information from the line *d*_1_ to *d*_3_, from *d*_3_ to *d*_5_, etc (it is possible to ignore *d*_2_, *d*_4_, …). In the artificial case, something similar happens as the blue arrows connect *r*_1_ with *r*_2_, *r*_2_ with *r*_3_, etc. There is a geometric transformation that maps the ordering of arrows in the biological case to the ordering of the artificial case (see Fig 6). This intuitively shows that the dynamics of both cases will be the same.

**Theorem (Proof in Section A.4 of S1 Text)**. Let the matrices be *M*_*FF*_ (feedforward weights) and $M_{FB}^{\left(q\right)}$ (feedback connections of distance *q*) in $R^{N\times N}$. If $M_{B}=M_{FF}+M_{FB}^{\left(q\right)}$ and $M_{A}={\left(Id-M_{FF}\right)}^{-1}M_{FB}^{\left(q\right)}$, then the characteristic polynomial of *M*_*B*_ and *M*_*A*_ can be expressed as:

$$\begin{array}{c} p_{A}\left(\lambda \right)=\lambda^{k_{1}}g\left(\lambda \right),\;p_{B}\left(\lambda \right)=\lambda^{k_{2}}g\left(\lambda^{k_{3}}\right) \end{array}$$ (9)

where *g* is a polynomial with coefficients that are functions of matrices *M*_*FF*_ and $M_{FB}^{\left(q\right)}$; $k_{1},k_{2}\in N_{0}$ and $k_{3}\in N$. In Table A in S1 Text, some examples of the polynomials *p*_*A*_ and *p*_*B*_ are shown. Note that the order of *g* and the integers *k*_1_, *k*_2_, *k*_3_ only depend on *N* and *q*.

An immediate consequence of the above Theorem is that if *M*_*B*_ has an eigenvalue with absolute value equal to 1, then *M*_*A*_ has an eigenvalue with absolute value equal to 1. To see this, choose an eigenvalue *z* of *M*_*B*_ (i.e. *p*_*B*_(*z*) = 0) with |*z*| = 1. Then $w=z^{k_{3}}$ will also satisfy *p*_*A*_(*w*) = 0 and |*w*| = 1. For this reason, *for a network with feedforward connections and feedback connections of a single distance q, the bifurcation boundaries of the artificial and biological implementation coincide*. Therefore, the dynamics of the implementation of networks with artificial transmission (Δ = 0) is equivalent to biological transmission (Δ = 1). This means that both have the same fixed points with the same stability region. This property allows replacing the biological implementation with the artificial implementation, which is *q* + 1 times less computationally expensive in terms of time and the number of operations.

An example of this result is shown in Fig 5b where *N* = 3 and *κ*_3_ = 0 (there are no *feedback connections of distance 2*). When *w*_*i*_ = *α*_11_ = *α*_22_ = *α*_33_ = 0, there is only *feedback of distance q* = 1; whereas when *w*_*p*_ = *κ*_2_ = 0, there are only *feedback of distance q* = 0 (see Table 1). In both cases, the stability region is the same for the biological and artificial implementations. In this particular example, the regions of stability coincide even when there are two types of feedback simultaneously (*q* = 0 and *q* = 1). But in general, the equivalence in stability between biological and artificial connections is only true if there is a single feedback distance *q* in the network. As we will discuss below, there are cases where considering mixed-feedback favors stability of the dynamics, and therefore the biological implementation is preferred in terms of stability.

### 3.3 Longer loops are more stable

To see the advantage of distant feedback, consider a simplified network such that all feedforward connections have the same weight (i.e. (*M*_*FF*_)_*i*,*j*_ = *βδ*_*j*,*i*−1_) and *feedback connections of distance q* only, all with the same weight (i.e. (*M*_*FB*_)_*i*,*j*_ = *fδ*_*j*,*i*+*q*_, |*f*| < 1). As seen in Section 3.2, the stability based on *M*_*A*_ is equivalent to the stability based on *M*_*B*_. For this simplified network, (*M*_*A*_)_*ij*_ = *βf*^*i*−*j*+*q*^Θ(*q* + 1 ≤ *j* ≤ *i* + *q*). That is,

$$\begin{array}{c} M_{A}=[\begin{array}{cc} 0 & *\\ 0 & L \end{array}] \end{array}$$ (10)

where the null blocks have *q* columns and the block $L\in R^{N-q\times N-q}$ is

$$\begin{array}{l} \;1\;\ldots \;q-1\;q\;q+1q+2\;\ldots \;N-q\\ L=\begin{array}{cc} \left[\begin{array}{cccccccc} \beta f^{q} & \ldots & \beta f^{2} & \beta f & \beta & 0 & \ldots & 0\\ \beta f^{q+1} & \ldots & \beta f^{3} & \beta f^{2} & \beta f & \beta & \ldots & 0\\ \ldots & \ldots & \ldots & \ldots & \ldots & \ldots & \ldots & \ldots \\ \ldots & & & & & & \beta f & \beta \\ \ldots & & & & & & \beta f^{2} & \beta f\\ \ldots & \ldots & \ldots & \ldots & \ldots & \ldots & \ldots & \ldots \\ \beta f^{N-1} & & & & \ldots & & \beta f^{q+1} & \beta f^{q} \end{array}\right] & \begin{array}{c} 1\\ 2\\ \ldots \\ N-2q\\ N-2q+1\\ \ldots \\ N-q \end{array} \end{array} \end{array}$$ (11)

A more compact way of writing *L* is

$$\begin{array}{l} \;1\;\ldots \;q-1\;q\;q+1\;q+2\;\ldots \;N-q\\ L=\beta [\begin{array}{cccccccc} f^{q}{\vec{c}}_{1} & \ldots & f^{q}{\vec{c}}_{2} & f{\vec{c}}_{1} & {\vec{c}}_{1} & \;{\vec{c}}_{2} & \ldots & {\vec{c}}_{N-2q} \end{array}] \end{array}$$ (12)

where ${\vec{c}}_{i}=\frac{{\vec{c}}_{i-1}-{\vec{e}}_{i}}{f}$ and

$$\begin{array}{cc} {\vec{c}}_{1} & =[\begin{array}{c} 1\\ f\\ \vdots \\ f^{N-q-1} \end{array}]. \end{array}$$

From the form of the matrix *M*_*A*_, it follows that there are at least *q* independent eigenvectors associated with the eigenvalue λ = 0. The other *N* − *q* eigenvalues correspond to those of the matrix *L*.

On the other hand, assuming $\vec{x}\in R^{N-q}$ one can show that

$$\begin{array}{c} L\vec{x}=\beta {\vec{c}}_{1}\sum_{i=1}^{N-q}x_{i}\;f^{q+1-i}-\beta \sum_{i=2}^{N-2q}\sum_{k=1}^{i-1}x_{i+q}{\vec{e}}_{i-k}\frac{1}{f^{k}}. \end{array}$$ (13)

**Fig 7:**
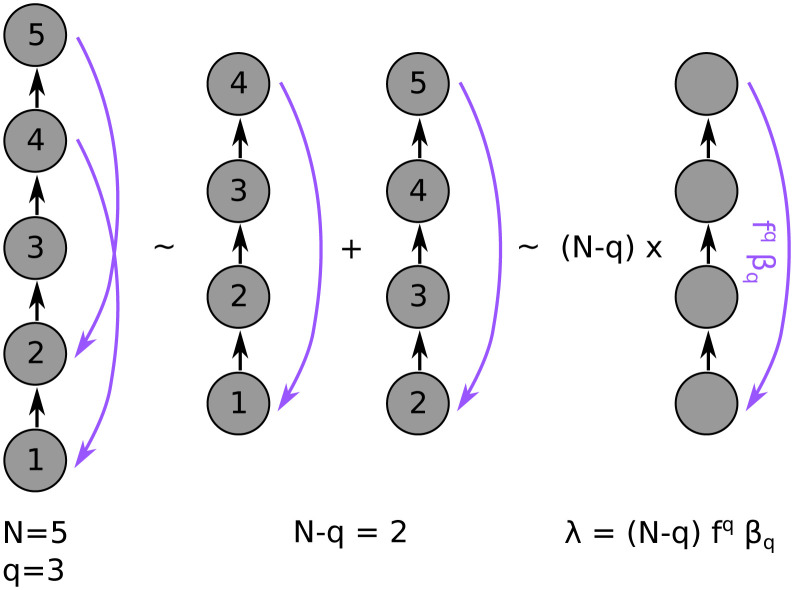
The dominant eigenvalue for a network with feedforward connections and feedback connections of a single distance *q*. The eigenvalue in this network is proportional to the number of loops and the effective weight of the loops (*f*^*q*^*β*_*q*_). When all feedback connections of distance *q* are considered, there are a total of *N*−*q* loops.

There are two cases. The first case is *N* − 2*q* < 2 (i.e. $\frac{N}{2}-1<q$). In this case, the equation $L\vec{x}=0$ has *N* − *q* − 1 independent solutions (i.e. *L* has *N* − *q* − 1 eigenvectors associated with the eigenvalue λ = 0). In addition, we find that ${\vec{c}}_{1}$ is an eigenvector of *L* associated with the eigenvalue λ = *βf*^*q*^(*N* − *q*). In this case, the stability of the network depends only on the factor *βf*^*q*^(*N* − *q*). The term of *βf*^*q*^ corresponds to the effective gain of one of the loops of distance *q*, while *N* − *q* is the number of loops of distance *q* in the network (see Fig 7). So, the stability condition is that the absolute value of the effective gain of the loops is less than $\frac{1}{N-q}$. Note that if the loops are longer (i.e. *q* increases), the number of loops *N* − *q* and the effective weight *f*^*q*^*b*_*q*_ decrease; then, the stability threshold increases. Even when the number of loops is predetermined (i.e. it does not depend on *q*), the term *f*^*q*^ continues to decrease as a function of *q* and modifies the threshold. This tells us that *networks with longer loops are more stable than with shorter loops*.

For the second case 2 ≤ *N* − 2*q* (i.e. $q\leq \frac{N}{2}-1$), the same result is obtained but using another argument. Note that in this case, the matrix *L* has *N* − 2*q* independent rows (i.e. the rank of *L* is *N* − 2*q*). Therefore, the dimension of the null space of *L* is *q* (i.e. the eigenvalue 0 has at least multiplicity *q*). This implies that

$$\begin{array}{c} p_{A}\left(\lambda \right)=\lambda^{2q}g\left(\lambda \right) \end{array}$$

where *g* is a polynomial of degree *N* − 2*q* whose roots satisfy

$$\begin{array}{c} \sum_{i=1}^{N-2q}\lambda_{i}=Tr\left(L\right)=\beta f^{q}\left(N-q\right). \end{array}$$ (14)

If λ_*i*_ > 0, then *max*(λ_*i*_) = *βf*^*q*^(*N* − *q*) defines the stability as in the previous case.

### 3.4 Fully connected networks are less stable

To see the advantages of a network with layers, consider a simple counter-example, a fully connected network with the connectivity matrix

$$\begin{array}{c} M_{B}=[\begin{array}{cccc} w_{i} & w_{e} & \ldots & w_{e}\\ w_{e} & w_{i} & \ldots & w_{e}\\ \vdots & & & \vdots \\ w_{e} & w_{e} & \ldots & w_{i} \end{array}]\in R^{N\times N}. \end{array}$$

**Fig 8:**
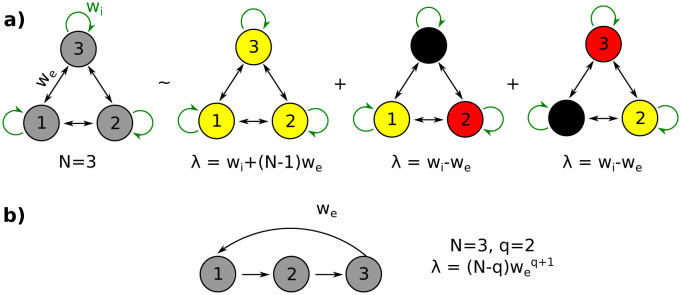
Fully connected and layered networks. a) Decomposition into eigenvalues and eigenstates of a fully connected network. Nodes of the same color are in-phase synchronized, the nodes with opposite colors (yellow-red) are anti-phase synchronized, and the black nodes are deactivated. b) Dominant eigenvalue in a layered network.

The weight of connections between units is *w*_*e*_ and the self-interaction weight is *w*_*i*_ (usually, *w*_*i*_ < 0). The eigenvalues of *M*_*B*_ are *w*_*i*_ − *w*_*e*_ (with multiplicity *N* − 1) and *w*_*i*_ + (*N* − 1)*w*_*e*_ (multiplicity 1) (see Fig 8a). In this case, the stability condition (i.e. eigenvalues with absolute value less than 1) is equivalent to $-\frac{1+w_{i}}{N-1}<w_{e}<\text{min}\;\left(w_{i}+1,\frac{1-w_{i}}{N-1}\right)$. In the limit of *N* → ∞, this region of stability converges to −1 < *w*_*i*_ < 0 and $|w_{e}|<\frac{1}{N-1}$. Note that the region of stability decreases for larger networks and does not depend on the distance of the feedback connections. Furthermore, the threshold of |*w*_*e*_| (i.e. $\frac{1}{N-1}$) is less than the threshold of |*w*_*e*_| in layered network with feedback connections of distance *q* (i.e. ${\left(\frac{1}{N-q}\right)}^{\frac{1}{q+1}}$) for all *q* (see Fig 8b). This would indicate that *networks ordered in layers are more stable than a fully connected network*. This is a consequence of the fact that in a fully connected network, all distances of the feedback appear, including the short distances that are the least stable.

### 3.5 Mixed-feedback

In the previous sections, we obtained that: (1) the area of stability increases as feedback distance *q* increases and (2) fully connected networks tend to be more unstable. An intermediate case is a network with feedback of two distances, say *q*_1_ < *q*_2_. We can calculate *p*_*B*_(λ) according to the proof of the theorem in Section A.4 of S1 Text. For the case that $\frac{N}{2}-1<q_{1}<q_{2}$, the relevant eigenvalue is

$$\begin{array}{c} \lambda =K_{1}^{\left(q_{1}\right)}+K_{2}^{\left(q_{1}\right)}+K_{1}^{\left(q_{2}\right)}+K_{2}^{\left(q_{2}\right)}∼\beta_{q_{1}}\;f^{q_{1}}\left(N-q_{1}\right)+\beta_{q_{2}}\;f^{q_{2}}\left(N-q_{2}\right). \end{array}$$

The region of stability in terms of $\beta_{q_{1}}\;f^{q_{1}}$ is:

$$\begin{array}{c} -\frac{1}{N-q_{1}}-\frac{N-q_{2}}{N-q_{1}}\beta_{q_{2}}\;f^{q_{2}}\leq \beta_{q_{1}}\;f^{q_{1}}\leq \frac{1}{N-q_{1}}-\frac{N-q_{2}}{N-q_{1}}\beta_{q_{2}}\;f^{q_{2}} \end{array}$$ (15)

For finite networks, the term due to the feedback with longer distance *q*_2_ helps to stabilize the dynamic. However, this effect is lost for very deep networks (very large *N*) since the stability region is defined by the equation $\frac{\beta_{q_{1}}}{\beta_{q_{2}}}=f^{q_{2}-q_{1}}$. Then, *adding a longer feedback connection favors the stability of the dynamics*.

### 3.6 Nonlinear dynamics

Thus far we present an analysis of the linear dynamics of a neural network, focusing on the eigenvalues of the matrices *M*_*B*_ = *M*_*FF*_ + *M*_*FB*_ and *M*_*A*_ = (*Id* − *M*_*FF*_)^−1^*M*_*FB*_ for both biological and artificial cases, respectively. In the presence of a nonlinear activation *F* in the system, the stability analysis around the fixed point relies on calculating the eigenvalues of the following matrices

$$\begin{array}{cc} M_{B}^{NL} & =Diag\left[F^{\prime }\left(I^{*}\right)\right]M_{B}\\ M_{A}^{NL} & ={\left(Id-Diag\left[F^{\prime }\left(I^{*}\right)\right]M_{FF}\right)}^{-1}Diag\left[F^{\prime }\left(I^{*}\right)\right]M_{FB} \end{array}$$

where

$$\begin{array}{c} {\vec{I}}^{*}=x{\vec{e}}_{1}+\left(M_{FF}+M_{FB}\right){\vec{h}}^{*} \end{array}$$ (16)

and ${\vec{h}}^{*}$ is the fixed point that satisfies ${\vec{h}}^{*}=F\left({\vec{I}}^{*}\right)$.

Table 2 provides an overview of the properties of several popular activation functions. Notably, while certain activation functions like sigmoid have bounded ranges, others like softplus have unbounded ranges. However, all these functions share the common feature of having bounded derivatives denoted by *F*′. Moreover, except the GELU and Sigmoid Linear functions, their derivatives are bounded within the interval [−1, 1]. Consequently, we can assert that ||*Diag*[*F*′(I*)]|| ≤ 1 and the eigenvalues of $M_{B}^{NL}$ and $M_{A}^{NL}$ are guaranteed to possess smaller absolute values compared to those of *M*_*B*_ and *M*_*A*_, respectively. As a result, the use of these nonlinear functions inherently promotes stability around the existing fixed point, making them favorable choices in neural network applications.

**Table 2:** Properties of activation functions.

Name	Function *F*	Range of *F*	Derivative *F*′	Rangeof *F*′
Sigmoid	$\sigma \left(x\right)=\frac{1}{1+e^{-x}}$	(0, 1)	*σ*(*x*)(1 − *σ*(*x*))	(0, 1)
H Tangent	2*σ*(2*x*) − 1	(0, 1)	4*σ*′(2*x*)	(0, 1)
Gaussian	$e^{-x^{2}}$	(0, 1]	$-2xe^{-x^{2}}$	[−0.86, 0.86]
ReLU	*x*Θ(*x*)	[0, ∞)	Θ(*x*)	{0, 1}
PReLU	*x*(*α*Θ(−*x*) + Θ(*x*))	(−∞, ∞)	*α*Θ(−*x*) + Θ(*x*)	{*α*, 1}
Sigmoid Linear	*xσ*(*x*)	[0, ∞)	*xσ*′(*x*)+ *σ*(*x*)	[−0.1, 1.1]
GELU	*x*Φ(*x*)	(−0.17, ∞)	*x*Φ(*x*) + Φ′(*x*)	[−0.12, 1.12]
ELU	$\left\{ \begin{array}{cc} \alpha (e^{x}-1) & x\leq 0\\ x & 0<x \end{array}\right.$	(−*α*, ∞)	$\left\{ \begin{array}{cc} \alpha e^{x} & x\leq 0\\ 1 & 0<x \end{array}\right.$	(0, 1]
SoftPlus	*ln*(1 + *e*^*x*^)	(0, ∞)	*σ*(*x*)	(0, 1)

An additional feature of some modern deep networks is the normalization of the input to the activation functions, such as batch normalization [33]. When batch normalization is included as part of a feedback loop it contributes to keeping the gain of that link in the feedback loop constant. Therefore, if the network starts with a stable configuration, normalization likely contributes to maintaining the overall feedback gain constant across training. It’s important to note that while there are arguments related to the transformation properties of batch normalization (e.g. linearity) that support this conjecture, even though a formal proof is still lacking.

### 3.7 Feedback connections improve detection of small objects

We will now use the recurrent CNN described in Section 2.2 to test the effects of feedback on the performance of object detection using a state-of-the-art architecture. Specifically, we implemented the Faster Region-CNN architecture (Faster R-CNN described in Section A.5 of S1 Text) with our recurrent CNNs as a *backbone*) and tested performance on the COCO dataset [34]. In this architecture, the backbone is trained to extract image features that serve the detection and classification of objects. We used various configurations of the recurrent CNN to test a range of layers and types of feedback in the backbone.

Our recurrent CNNs use the same stages (or parts of them) of the ResNet-50 (see Section A.6 of S1 Text). Thus, we were able to initialize our networks with the corresponding weights from the pretrained ResNet-50 [30], which we then fine-tuned on the COCO dataset. The implementation code and the configuration files for the networks used here are available at GitHub. We use 118k images for training and 5k images for testing. In both stages, there are 80 categories of objects to be detected.

**Fig 9:**
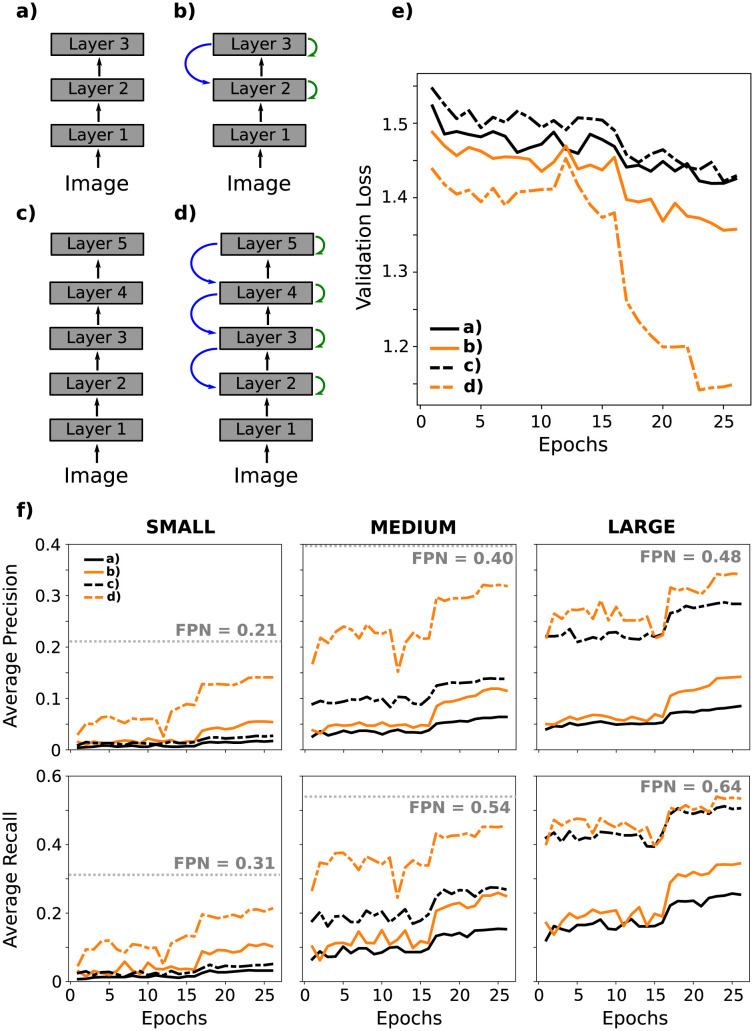
Performance on object detection during the training process. The results here are on the validation set. Recurrent CNNs (a-d) were used as backbones in Faster R-CNN. (a, c) Feedforward networks with 3 and 5 layers, respectively. (b, d) Feedback connections of distance 0 (green arrows) and 1 (blue arrows) were added to networks a) and c). (e) During the training stage, validation loss of the feedforward networks evolve similarly, regardless of depth (lines a), c)). Adding feedback connections reduce the validation loss (lines b), d)). (f) Average precision and recall for detection of objects of different sizes in images of the validation set. The initial value of each metric (epoch 1) tends to be higher as the number of layers in the network increases. However, the evolution of each metric depends on the size of the image and whether feedback connections are included. The gray line indicates the performance of the Feature Pyramidal Network (FPN) pretrained on this data [35].

We monitor the loss for the validation set during the training process for the four different backbones we tested (see Fig 9e). Two of the backbones are purely feedforward CNNs with 3 and 5 layers (see a),c) in Fig 9, respectively). The validation loss does not improve much during training and there is minimal benefit to increasing the number of layers from 3 to 5. We also tested the same networks, but now including feedback connections of distance 0 and 1 (see b),d) in Fig 9, respectively). In these latter cases, we use time delay Δ = 1 for the feedforward connections between layers (see Section 2.2). Artificial delays with Δ = 0 tended to become unstable during learning and were not further explored. For both networks, adding feedback reduced the validation loss. Adding feedback connections is better than adding layers with feedforward connections.

**Fig 10:**
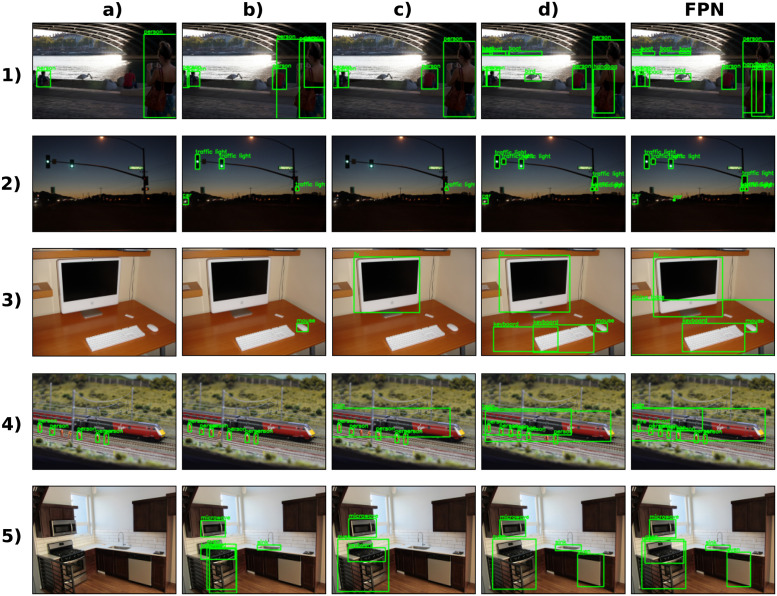
Examples of object detection and classification results. Predictions for five images (rows) of the evaluation set using Faster R-CNN. The backbone for Faster Region-CNN is one of four of our recurring CNNs or Feature Pyramidal Network—FPN (columns).

We also evaluated standard performance measures in this task, namely, the Average Recall (AR) and Average Precision (AP) on small, medium, and large objects, as defined in Section A.7 of S1 Text. The Faster R-CNN architecture proposed regions of interest based on the backbone output and then classifies or dismisses them. The results presented in Figs 9 and 10 are calculated using a maximum of *N*_*prop*_ = 100 region proposals per images and threshold values of intersection-over-union (*IoU*) in the range 0.5 : 0.95 (for more details, see Section A.7 of S1 Text).

Fig 9f presents the evolution of these metrics during training. Each column corresponds to the size of objects (small, medium, large). These results show that the initial and final values of the metrics, and their temporal evolution, depend on the depth of the network, the feedback connections, and the size of the objects. More precisely, we observed that the initial performance (epoch 1), for both AP and AR, is higher in deeper networks. This result is independent of the size of the objects (see Fig 9f) and is due to the fact that the networks c), and d) are more similar to the pretrained ResNet-50. In addition, when the network has five layers, the initial value of the metrics for large objects are not changed if feedback connections are added; however, for medium and small objects, these connections help to improve performance.

Note that networks with feedback connections perform better than feedforward networks of the same depth (compare black vs orange lines in Fig 9). This result is the same for all sizes of objects. Furthermore, for small objects, the network with three layers and feedback connections has better performance than the five-layer network without feedback connections (orange solid lines vs black dashed lines).

For comparison Fig 9 also show the performance of the Feature Pyramidal Network, which is a current benchmark for this object detection task [35]. The FPN architecture consists of a bottom-up pathway, a top-down pathway, and lateral connections. As in our architectures, the bottom-up pathway is the feedforward computation of the backbone (i.e. *F*_*L*_ in Eq (6)). More precisely, [35] uses a ResNet-50. The main difference between FPN and our architectures is the implementation of the feedback (i.e. recurrent map *R* in Eq (4) and integration mechanism *ϕ*_*l*_ in Eq (5)). The FPN can be thought of as having feedback from all layers (hence the name “feature pyramid”) and recurrence is iterated for a single time step. It is likely this hierarchical feedback that provides a performance boost to the FPN.

In Fig 10, we show some examples of predictions with the Faster Region-CNN using different backbones. In the first four columns (a-d), we are using the recurrent CNNs implemented here as the backbone (i.e. Fig 9(a)–9(d)); while in the last column, we use the FPN. The examples in Fig 10 show that for networks with feedback connections, the detection was improved over small and medium objects (see b) vs a) and d) vs c)). Furthermore, the predictions shown for the network d) and FPN coincide in most cases.

### 3.8 Feedback connections improve robustness against noise

**Fig 11:**
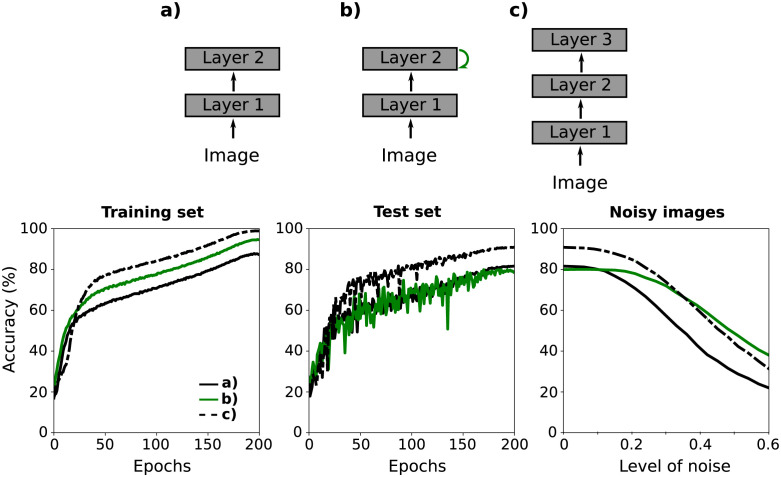
Accuracy in classification task. Recurrent CNNs (a-c) were used as feature extractors in the classification task. (a, c) Feedforwards networks with 2 and 3 layers, respectively. b) Feedback connection of distance 0 (green arrow) was added to network a). During the training of the networks (a-c), the accuracy calculated over the training set and test set increases. The performance of the networks is reduced when evaluating on the images of the test set with Gaussian noise.

In this section, we discuss the effect of feedback on image classification performance. For this, we implement a neural network that consists of a features extractor, a pooling operation, and a classifier (i.e. perceptron). We use three networks presented in Fig 11(a)–11(c) as feature extractors. Networks a) and c) are feedforward architectures with two and three layers, respectively. Also, only one distance feedback connection *q* = 0 was added to the network a) (see Fig 11b). As in the previous section, for the feature extractors (a-c), we use the architecture described in Section 2.2, but using ResNet-18 stages.

We use the CIFAR-10 dataset which consists of 60000 color images in 10 classes (0: airplane, 1: automobile, 2: bird, 3: car, 4: deer, 5: dog, 6: frog, 7: horse, 8: ship, 9: truck), with 6000 images per class. There are 50000 training images and 10000 test images [36]. The output of the classifier is a 10-dimensional vector indicating the probability that the input image belongs to each class and the final prediction of the network is the class with the highest probability. The evaluation of the networks is expressed in terms of accuracy and its confidence intervals (95%), which were estimated using a bootstrap procedure.

In Fig 11, we show the accuracy of the three networks calculated for different groups of images. In the left and center panels, accuracy as a function of training epochs was calculated using the training set and test images, respectively. After training, the deepest network (c) performs better than the other two (test acc = 90.2 ± 0.3%). Note that while network (b) fits the training data better than (a) (94.1 ± 0.3% vs 87.2 ± 0.4%), network (a) performs better over the test set (81.3 ± 0.4% vs 78.2 ± 0.4%). In the right panel, the performance of trained networks on noisy images is shown. Gaussian noise was added to each image in the test set. The noise has a mean value of 0 and the standard deviation is proportional to the standard deviation of the dataset. This proportionality factor is called “noise level”. Note that the performance of network (b) is higher than that of network (a) when the noise level is greater than 0.1. Furthermore, it is also greater than the performance of the network (c) when the noise level is greater than 0.35. That is, from a certain noise level, the performance of the recurrent network (b) will be more robust against noise than that of the purely feedforward networks (a,c).

### 3.9 Activity dynamic reduces entropy, improving classification performance over time

Due to the dynamic activity of the network, the output of the classifier (i.e. vector of probabilities) changes over time during the inference stage. Furthermore, each vector is associated with an entropy value (i.e. -$\sum_{i=0}^{9}p_{i}\;{\text{log}}_{2}\;p_{i}$). In cases where entropy is high (∼ log_2_ 10 = 3.13), all probabilities are close to $\frac{1}{10}$, indicating that the network is less certain about a class selection. Conversely, in cases of low entropy (∼ 0), there is a class with a maximum probability close to 1. Consequently, in high-entropy cases, the final prediction is more susceptible to errors.

**Fig 12:**
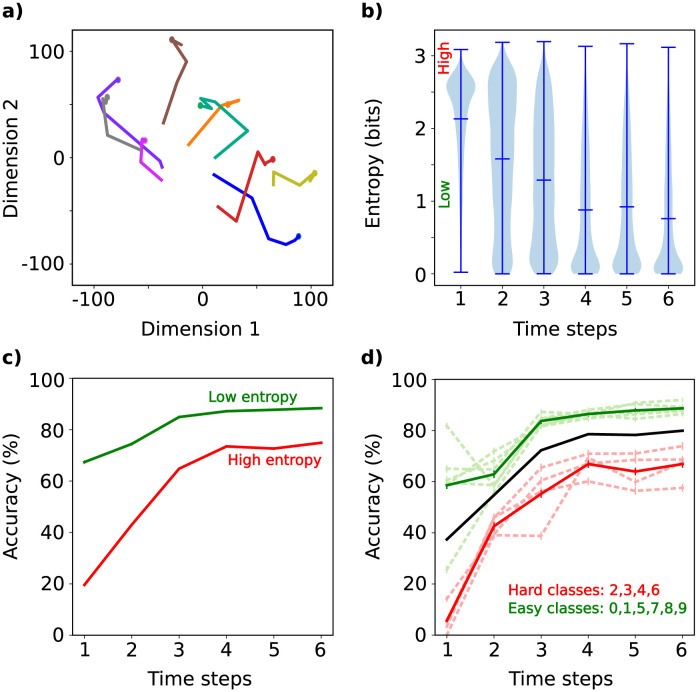
Temporal dynamic of the classification network. This simulation uses the trained network of Fig 11b. a) Examples of t-SNE projection of the trajectories of the activity space of the last layer. The activity space has dimension 10; while the projection is two-dimensional. Please note that for feedforward networks without temporal dynamics, the trajectory is a point that remains constant over time. Therefore, the stability of the dynamics during inference is assured. b) Distribution of entropy of the output as a function of the time steps in the inference stage. c) Performance of the network as a function of the time steps in the inference stage for the low (green line) and high (red line) entropy images. d) Performance of the network as a function of the time steps in the inference stage. We evaluated the network on the complete test (solid black line) and by classes (dashed lines). Based on the accuracy by classes, the easy and hard classes to classify by the network were identified (green and red lines, respectively).

For the trained network with the feature extractor shown in Fig 11b, we computed the classifier’s output for all images in the test set and for different time steps of the activity dynamic (*t* = 1, …, 6). We applied the t-SNE visualization method [37] to show the temporal evolution of the output vector in the activity space for some input images (Fig 12a). Notably, these trajectories converge to a fixed point, replicating the schematic representation of Fig 4b. When monitoring the entropy of outputs, we find that the dynamic decreases in uncertainty as to the class identity over time, i.e. the networks gains in “confidence” over time (Fig 12b).

We categorized images with *high entropy* (resp. *low entropy*) as those whose outputs have entropy greater (resp. lower) than the mean entropy at *t* = 1 (see *t* = 1 in Fig 12b). In Fig 12c, we show the network’s performance as a function of time steps in the inference stage for both groups (red curve: *high entropy*, green curve: *low entropy*). The network’s performance outperforms in cases of *low entropy*, reaching a convergence of 88%, while for high entropy, it converges to 74% (see Fig 12c). The time needed for performance convergence differs between the two groups. Specifically, at *t* = 3, the low entropy group’s performance is 96% of the final value, whereas for the high entropy group, it’s 86%. The high entropy group requires an additional time step (*t* = 4) to reach 96% of the final value. This result is reminiscent of the finding in the inferior temporal (IT) cortex of primates, whereby neurons reach more “confident” decisions later in time (∼ 30 ms) for more challenging images (see Fig 2 in [18]). We see a similar result (Fig 12d) when separating performance for classes that are more challenging to identify (class 2: bird, class 3: car, class 4: deer, and class 6: frog).

## 4 Discussion

In this work, we studied the dynamics of recurrent networks with static inputs. We observed that the stability region for networks with biologically realistic feedforward delay is larger than for artificial networks without feedforward delays. Furthermore, we showed that in networks with feedback connections of fixed distance, the stability of both implementations (biological and artificial) are equivalent. This is a consequence of the presence of a single time scale when only a single feedback distance is present. Using this last result, we found that the effective gain of longer loops dominates the dynamic and improves overall stability. In fact, adding longer distance loops can improve the stability of a recurrent network. Note that, implicitly, deeper networks can accommodate longer loops. Furthermore, layered networks tend to be more stable than fully connected networks, as they tend to increase the loop distance compared to fully connected networks. While some of these mathematical results were derived with “layers” consisting of individual units, we showed that the results generalize to layers with multiple uniform units, which is common in both artificial and biological recurrent networks. Finally, we demonstrated that typically nonlinear activation functions only contributed to increase stability. In total, we found that basic organizational principles in biological networks favor stability, namely, feedforward delays, a layered organization with similar units in each layer, long-range feedback, and nonlinear activations.

The computational power of deep networks has now been widely demonstrated, with state-of-the-art performance using up to a hundred or more layers. However, such very deep networks are not biologically realistic, and the argument has been made that recurrent processing can add processing steps in a reduced architecture [38, 39]. Therefore, the important question is whether adding feedback benefit performance at a limited depth. We implemented and evaluated recurrent CNNs for object detection and image classification in the COCO and CIFAR10 datasets, respectively. We used biological feedback to ensure stability during learning. The feedback connections helped to improve the detection of small objects and to obtain robust performances against noise in the classification task. This is consistent with previous work [16, 40, 41] showing that recurrent dynamics improve recognition performance in the challenging scenario of partial occlusion (e.g., multiple targets occluding each other) or degraded images. Importantly, the temporal dynamics of these recurrent networks were reminiscent of the activity dynamic in biological vision [18] as discussed in more detail below.

The analytical results we derived here assumed a simplified linear recurrent network. For nonlinear networks, the same analysis can be carried out by linearizing around fixed points. As we showed in Section 3.6 the typical nonlinear activation functions used in current network models can only improve stability in existing fixed points. In this sense, here we performed as worst-case analysis. In nonlinear networks with bounded activations (as in biological systems), even unstable fix points are likely to result in oscillations with stable limit cycles. The analysis of such limit cycles is more complex and beyond the scope of this work. Another limitation of this work is that many of the analytic results were obtained for special cases with simplified connection weights that capture the essence of the phenomena. We conjecture that similar results hold on average under random connection strengths. Similarly, the results were derived for uniform time delays. However, in biological networks, time delays are not uniform across the network. An outlook on how to treat the case of non-uniform time delays is provided in Section A.1 of S1 Text.

From a mathematical perspective, Eqs (1) and (4) represent the temporal evolution of the activity in neural networks and are examples of discrete-time dynamic systems [29]. Some of the main results of this work are a consequence of applying the bifurcation theory of dynamic systems to these cases. The temporal evolution ${\vec{h}}_{t}$ depends on the weights of the network connections. The set of weights and the input *x* (i.e. image) define the possible trajectories that exist in phase space (see Fig 1c). That is, the structure of the phase space (e.g. fixed points, periodic orbits, invariant torus) will also depend on the parameters of the network. For fixed points of the dynamic, we studied its behavior as a function of the connectivity and identified the bifurcation point where its local stability changes. This type of stability analysis is one of the first steps in the general study of phase space. The next step is the analysis of attractors or limit cycles [29]. However, in our work, this step is sufficient as we have focused on vision tasks associated with static images, such as classification and object detection. These tasks are considered core vision that is completed in primate within a few hundred milliseconds [3, 18, 27, 28, 42], i.e. within a single fixation. The importance of feedback in the core vision has been demonstrated, for instance, in the classification of images in background clutter [18]. The last “layer” of this system of core vision is the inferior temporal (IT) cortex where one can linearly decode the class identity of images from neural activity. As time progresses after the image presentation, the decoding performance increases reaching a peak at 100–200 ms [18]. Importantly, challenging images take longer to “decode” by about 30 ms which corresponds to approximately two additional processing steps. Here we found that in object classification with top-down feedback, performance increases over time of the activity dynamic with challenging images taking longer to achieve maximum performance (Fig 12). A limitation of the present work is that we have only analyzed the case of a static input. Yet, primate vision is marked by static input during fixations, but changes of fixation in a sequence of saccades, often attracted by salient and moving objects. It would be interesting to determine the role of feedback in those dynamical contexts [43–45], where information across fixations is integrated.

In the context of time-sequence processing, a dynamic that converges to a fixed point may be quite restrictive, and a more diverse dynamic, perhaps with limit cycles, could be more expressive [46]. However, in the context of static inputs, we note that purely feedforward nonlinear networks can be highly expressive, despite being “stable”. Empirically, we found that adding loops to pretrained deep networks can enhance performance. The search began in the proximity of an expressive network and led to improvements. This leads us to conclude that there are situations where adding stable feedback can contribute to the expressiveness of nonlinear networks. In addition to fixed points and stable limit cycles, neural networks can exhibit chaotic behavior. Chaotic dynamics can be leveraged to enhance information processing capacity, long-term memory [47], and adaptability in practical applications [48]. However, chaotic dynamics can also introduce challenges in prediction, control, and training due to their extreme sensitivity to initial conditions [49]. This sensitivity must be carefully considered in the system design and the tuning of learning parameters to ensure stability and proper functionality.

Here, we argued that the structured organization of connections in a network contributes to stability. However, at first glance, the visual system seems to exhibit densely interconnected recurrent pathways [50]. It’s important to recognize, though, that brain networks are far from fully connected [51]. In particular, for the ventral visual pathway, there is clear sequential processing across the processing hierarchy with top-down feedback [12]. There are also connections of the visual hierarchy with subcortical brain nuclei and other cortices, but these are not necessarily reciprocal connections [12]. The simplified model structure we analyzed here (e.g. Fig 5c) is motivated by the specifics of the wiring diagram (Fig 1b) that have been proposed for core vision, e.g. [28]. Future work may use the formalism proposed here to analyze the stability of other network motives.

Here we emphasized stability when discussing network organization. There are a multitude of theoretical and experimental studies on other principles of network organization in the mammalian brain. On the largest scale of the whole brain, this includes observation of a small-world structure with densely connected hubs and sparse long-range connections [51]. The overall structure of the human brain appears to form a set of segregated networks that exhibit correlations within each network [52], such as the default mode network, ventral and dorsal attention networks, visual network, etc. Brain organization also appears to exhibit gradients in the microstructure such as inhibitory and excitatory strength and connectivity [53] as well as functional gradients such as in the time scale [54] which has been linked to cortical microstructure [55]. Here we have narrowly focused on the effect of delays on stability, and how different connectivity motives may aid stability, and contrasted this with how sequential processing in artificial neural networks incorporate delays. We found that layers and long-range feedback contribute to stability, but do not mean to imply that the only purpose of the layered organization is stability. Stability is also facilitated, for instance by a balance between excitation and inhibitory feedback, e.g. [56]. A caveat of the present study is that we have not analyzed this important principle of stability in biological networks [57].

As mentioned above, the fixed points do not depend on the number of time steps used. When a trajectory converges to a fixed point, it can be interpreted that this point condenses all the information of this trajectory. Therefore, each input of the network *x* and initial condition *h*_0_, will be associated with a fixed point ${\vec{h}}^{*}$. This interpretation seems to be very similar to the simple construction of a feedforward network (input *x*—output ${\vec{h}}^{*}$ relationship). However, the main difference is that each fixed point of a dynamic system defines a *basin of attraction*: small perturbations of *x* and *h*_0_ (e.g. Δ*x*: noise) do not modify the fixed point ${\vec{h}}^{*}$. We believe that this is the basis for the robustness of the dynamics against the noise we have observed. On the other hand, stable fixed points are a particular case of bounded dynamics (i.e. $|{\vec{h}}_{t}|<M$). Clearly, dynamic systems with unbounded trajectories are a serious problem both for the calculation of the output and the training of the network (calculation of gradients).

Although recurrent networks seem to be crucial in visual processing, a bottleneck for computational models is the computational cost of the standard algorithm for training (BPTT: “back-propagation through time”) [24], which has to propagate errors backward in time for every learning step. In recent years, efforts have focused on efficient approximations to BPTT [25, 58]. An example is recurrent backpropagation (RBP) which assumes that the dynamical system converges to a task-optimized fixed point; under this assumption, a constant O(1) memory-complexity is achieved with recursive processing steps. In [31], Linsley shows that stable dynamics improve performance in large-scale computer vision challenges. However, in general, this assumption is very strong as it depends on the network parameters during training. One way to ensure that recurrent models are stable is to apply penalties (e.g. Contractor-RBP) [31]. In this work, we have shown that both the architecture (i.e. type of connections and order of loops) and type of feedforward transmission (biological vs artificial) plays an important role in the stability of the dynamics. The results presented here indicate that there are more favorable architectures for the application of RBP, which may not require additional constraints to ensure stability. Specifically, one can show that RBP becomes an exact algorithm if the dynamic has a stable fix point (in preparation). In short, with the proper choice of feedback, deep learning models may become easily trainable biologically inspired networks.

A possible future direction of this research is to analyze the role of feedback connections at different levels. The most popular networks for visual processing tasks consist of 1) a backbone that reduces the dimensionality of the input using convolutions and returns a set of features, and 2) a predictor that returns the output as a function of the features. In the architectures presented in this work, we only added feedback connections to the backbone (low/mid-level); while the predictor was not modified. Some works use architectures where only the predictor is a recurrent network [59, 60]. The next step is to use both levels of feedback connections, which may represent the top-down feedback across fixations and can be useful for the integration of information across a larger image (e.g. interaction between objects, action recognition) or may serve to integrate information across time in a dynamic visual input (e.g. video processing).

## Supporting information

S1 Text Mathematical proofs and implementation details. (PDF)
