# Supplementary material for: Architecture of the brain’s visual system enhances network stability and performance through layers, delays, and feedback
Source: PLoS Comput Biol. 2023 Nov 10;19(11):e1011078. doi: 10.1371/journal.pcbi.1011078 (PMC10664920; doi:10.1371/journal.pcbi.1011078)
Supplement: S1 Text — (PDF) [file pcbi.1011078.s001.pdf]

Architecture of the Brain's Visual System Enhances Network Stability and Performance Through Layers, Delays, and Feedback

Osvaldo Matias Velarde[1,*], Hernán A. Makse[2], Lucas C. Parra [1]

1 Biomedical Engineering Department, The City College of New York, New York, New York, United States of America
2 Levich Institute and Physics Department, The City College of New York, New York, New York, United States of America

*** ovelarde@ccny.cuny.edu**

A Supporting information

A.1 Fully connected networks and variable time delays

Given the graph $\mathcal{G}$ that defines the connectivity of a neural network, the notions of feedback, feedforward, and skip connections are meaningful only if there exists a unique directed path $\mathcal{C}_{FF}$ from the input node to the output node

$$\mathcal{C}_{FF} = \arg\min_{\mathcal{C}} \sum_{c \in \mathcal{C}} \Delta_c \tag{1}$$

where $c \in \mathcal{C}$ indicates a connection c is part of the path $\mathcal{C}$.

The nodes along the path C_{FF} are referred to as *layers* and are indexed as $l \in 1, ..., N$, where $l = 1$ represents the input node, and $l = N$ represents the output node. Within this framework, the definitions of *feedforward* and *feedback* connections align with those presented in Section 2. For the architecture we analyzed in Section 3.4 the feedforward path C_{FF} is well-defined. However, in a fully connected network with connections having the same temporal delay Δ, the path C_{FF} is not well-defined, and the analysis of Section 3.4 loses meaning as there is no notion to distinguish feedforward from feedback connections.

We have assumed uniform delay in all connections. However, in biological networks delays may differ, with the shortest delays typically appearing within layers. If we take these shortest delays as the unit of time and express all other delays as multiples, then the analysis we have carried out here could be extended to non-uniform delays by analyzing stability using the Z-transform, following [1]. If discrete delays are insufficient, then one would have to switch to continuous dynamical systems approximations. In both cases, the stability analysis involves finding the solutions to the characteristic equation. In the continuous approximation, the characteristic equation is typically a transcendental equation, unlike the case with a single delay where the characteristic equation is a polynomial equation (Section 2.1). This may be the subject of future work.

A.2 Stability analysis for layers with multiple units.

In Section 3.1, we analyze the stability for networks with N layers with a single unit each. For this reduced network, let us denote M_{FF}^{red} and M_{FB}^{red} to be the weight matrices of the feedforward and feedback connections, respectively. Also, let $\Lambda_{B,red}$ and $\Lambda_{A,red}$

be the sets of eigenvalues for the matrices $M_B^{red} = M_{FF}^{red} + M_{FB}^{red}$ and $M_A^{red} = (Id - M_{FF}^{red})^{-1} M_{FB}^{red}$ corresponding to the biological and artificial implementations, respectively.

Let's now assume a network with m units per layer. A unit i of layer l can project onto any unit of layer $l+1$ with weight $\alpha_{l \to l+1}$ (feedforward connections). On the other hand, units in the same layer do not interact, and feedback connections between layers $l_1 \to l_2$ will be topographic, namely, they only affect units in the same position i, say, with weight $\alpha_{l_1 \to l_2}$. These assumptions can be summarized in the following connection weight matrices M_{FF} and M_{FB},

$$M_{FF} = \mathbf{1}_{m\times m} \otimes M_{FF}^{red} \in \mathbb{R}^{N.m}$$
$$M_{FB} = \mathbf{1}_{m\times m} \otimes M_{FB}^{red} \in \mathbb{R}^{N.m}$$

where $\mathbf{1}_{m\times m} \in \mathbb{R}^m$ is the matrix with all entries equal to 1 and $\otimes$ indicates the tensorial product.

For this extended network, the matrices associated with the biological and artificial implementations are:

$$M_B = M_{FF} + M_{FB} = \mathbf{1}_{m\times m} \otimes M_B^{red} \tag{2}$$

and

$$\begin{aligned}
M_A &= (Id - M_{FF})^{-1} M_{FB} \\
&= \sum_{k=0}^{\infty} M_{FF}^k M_{FB} \\
&= \sum_{k=0}^{\infty} (\mathbf{1}_{m\times m} \otimes M_{FF}^{red})^k (\mathbf{1}_{m\times m} \otimes M_{FB}^{red}) \\
&= \sum_{k=0}^{\infty} (\mathbf{1}_{m\times m}^k \otimes M_{FF}^{red(k)}) (\mathbf{1}_{m\times m} \otimes M_{FB}^{red}) \\
&= \left[Id + \sum_{k=1}^{\infty} (m^{k-1} \mathbf{1}_{m\times m} \otimes M_{FF}^{red(k)}) \right] (\mathbf{1}_{m\times m} \otimes M_{FB}^{red}) \\
&= \left[Id + \frac{1}{m} \sum_{k=1}^{\infty} (\mathbf{1}_{m\times m} \otimes (m M_{FF}^{red})^k) \right] (\mathbf{1}_{m\times m} \otimes M_{FB}^{red}) \\
&= \left[Id + \frac{1}{m} (\mathbf{1}_{m\times m} \otimes \sum_{k=1}^{\infty} (m M_{FF}^{red})^k) \right] (\mathbf{1}_{m\times m} \otimes M_{FB}^{red}) \\
&= \left[Id + \frac{1}{m} (\mathbf{1}_{m\times m} \otimes ((Id - m M_{FF}^{red})^{-1}) - Id)) \right] (\mathbf{1}_{m\times m} \otimes M_{FB}^{red}) \\
&= \mathbf{1}_{m\times m} \otimes ((Id - m M_{FF}^{red})^{-1} M_{FB}^{red})
\end{aligned}$$

Note that the eigenvalues of $\mathbf{1}_{m\times m}$ are 0 (multiplicity $m-1$) and m (multiplicity 1). Then, using properties of the tensor product, the eigenvalues of M_B become 0 and the eigenvalues of M_B^{red} are multiplied by m. On other hand, the eigenvalues of M_A are 0 and the eigenvalues of the matrix $(Id - m M_{FF}^{red})^{-1} M_{FB}^{red}$ multiplied by m. It is important to note that the eigenvalues of $(Id - m M_{FF}^{red})^{-1} M_{FB}^{red}$ are not equal to those of $M_A^{network}$ but are at least m-times larger. Therefore, the eigenvalues of M_A are m^2 larger than those of M_A^{red}.

As mentioned above, the bifurcation boundaries for extended networks are the result of applying a scale factor to those of reduced networks. The scale factors are $\frac{1}{m}$ and $\frac{1}{m^2}$

for the biological and artificial cases, respectively. From the analysis presented in 3.1, we know that for reduced networks, the area of the region of stability of the biological implementation is greater than that of the artificial implementation. This fact does not change when applying the scale factors to the bifurcation boundaries. Essentially we find that the stability advantage of having feedforward delays also holds for networks with many units per layer, at least for a particular simple topographic feedback structure.

A.3 Roots of Polynomial with absolute value equal to 1

Let the polynomial $p(\lambda) = \sum_{n=0}^{N} c_n \lambda^n$ and $\theta \in [0, 2\pi)$ such that $p(e^{i\theta}) = 0$. Separating the real and imaginary parts of the equation, we obtain:

$$0 = \sum_{n=0}^{N} c_n \cos(n\theta) = \sum_{n=0}^{N} c_n T_n(z)$$
$$0 = \sum_{n=1}^{N} c_n \sin(n\theta) = \sum_{n=1}^{N} c_n \sin(\theta) U_{n-1}(z)$$

where $z = \cos(\theta)$, T_n's and U_n's are Chebyshev polynomials of the first and second kind, respectively [2].

The equality is satisfied for two cases. The first case is when $\theta = m\pi$ (i.e. $\lambda = \pm 1$) and it happens when the coefficients c_n satisfy $0 = \sum_{n=0}^{N} c_n (-1)^{mn}$. The other case is when the following equations are fulfilled simultaneously

$$0 = \sum_{n=1}^{N} c_n U_{n-1}(z)$$
$$0 = \sum_{n=0}^{N} c_n T_n(z)$$

Using the relation $T_n(z) = U_n(z) - zU_{n-1}(z)$ in the last equation:

$$\begin{aligned}
0 &= \sum_{n=0}^{N} c_n T_n(z) \\
&= c_0 T_0(z) + \sum_{n=1}^{N} c_n [U_n(z) - zU_{n-1}(z)] \\
&= \sum_{n=0}^{N} c_n U_n(z) \\
&= c_0 + c_1 U_1 + \sum_{n=2}^{N} c_n [2zU_{n-1} - U_{n-2}] \\
&= c_0 + c_1 U_1 - 2zc_1 U_0 - \sum_{n=0}^{N-2} c_{n+2} U_n \\
&= c_0 - \sum_{n=0}^{N-2} c_{n+2} U_n.
\end{aligned}$$

Then, the parameter conditions (i.e. bifurcation boundaries) are:

$$0 = \sum_{n=0}^{N-1} c_{n+1} U_n(z), \quad c_0 = \sum_{n=0}^{N-2} c_{n+2} U_n(z).$$

A.4 Demostration of Theorem

First, let's calculate the inverse of the matrix $-\lambda Id + M_{FF}$. Using properties of triangular matrices, we obtain

$$(-\lambda Id + M_{FF})^{-1}_{ij} = \begin{cases} 0 & i < j \\ -(\frac{1}{\lambda})^{i-j+1} \prod_{k=j}^{i-1} \alpha_{k,k+1} & j \leq i \end{cases} \tag{3}$$

We will calculate the polynomial $p_B(\lambda) = det(M_B - \lambda Id)$. Note that $M_B - \lambda Id = M_{FF} + M_{FB}^{(q)} - \lambda Id \in \mathbb{R}^{N \times N}$ has the form

$$M_B - \lambda Id = \left[\begin{array}{c|c} S_1 & S_2 \\ \hline S_3 & S_4 \end{array} \right] \tag{4}$$

where the blocks $S_1 \in \mathbb{R}^{q \times q}$, $S_2 \in \mathbb{R}^{q \times N-q}$, $S_3 \in \mathbb{R}^{N-q \times q}$ and $S_4 \in \mathbb{R}^{N-q \times N-q}$ are defined by:

$$\begin{aligned}
(S_1)_{ij} &= -\lambda \delta_{ij} + \alpha_{ji} \delta_{i,j+1} \\
(S_2)_{ij} &= \alpha_{q+j,j} \delta_{ij} \\
(S_3)_{ij} &= \alpha_{q,q+1} \delta_{i,1} \delta_{j,q} \\
(S_4)_{ij} &= -\lambda \delta_{ij} + \alpha_{q+j,q+i} \delta_{i,j+1} + \alpha_{q+j,j} \delta_{i,j-q}
\end{aligned}$$

Then, using the block matrix determinant formula, $p_B(\lambda) = det(S_1) det(S_4 - S_3 S_1^{-1} S_2)$. Since S_1 is a lower triangular matrix, $det(S_1) = (-\lambda)^q$. On the other hand, the product is

$$\begin{aligned}
(S_3 S_1^{-1} S_2)_{ij} &= \sum_{k,r} (S_3)_{ik} (S_1^{-1})_{kr} (S_2)_{rj} \\
&= \sum_{k,r} \alpha_{q,q+1} \delta_{i,1} \delta_{k,q} (S_1^{-1})_{kr} \alpha_{q+j,j} \delta_{rj} \\
&= \alpha_{q,q+1} \alpha_{q+j,j} (S_1^{-1})_{qj} \delta_{i,1} \\
&= f_j \delta_{i,1}
\end{aligned}$$

where $f_j = \alpha_{q,q+1} \alpha_{q+j,j} (S_1^{-1})_{qj}$. Also, $(S_1^{-1})_{ij}$ can be calculated using Eq. (3) and we obtain that $f_j = -(\frac{1}{\lambda})^{q-j+1} \alpha_{q+j,j} \prod_{k=j}^{q} \alpha_{k,k+1}$.

For now, we focus on the case $q > \frac{N}{2} - 1$. Then, calculating by columns, we obtained that

$$det(S_4 - S_3 S_1^{-1} S_2) = \begin{vmatrix} -\lambda - f_1 & -f_2 & -f_3 & ... & f_{N-q} \\ \alpha_{q+1,q+2} & -\lambda & 0 & ... & 0 \\ 0 & \alpha_{q+2,q+3} & -\lambda & ... & 0 \\ ... & ... & ... & ... & ... \\ 0 & & & \alpha_{N-1,N} & -\lambda \end{vmatrix}$$

$$\begin{aligned} &= (-\lambda - f_1)(-\lambda)^{N-q-1} + \sum_{j=2}^{N-q} (-1)^j f_j (-\lambda)^{N-q-j} \prod_{k=q+1}^{q+j-1} \alpha_{k,k+1} \\ &= (-\lambda - f_1)(-\lambda)^{N-q-1} - (-1)^{N-q} \lambda^{N-2q-1} \sum_{j=2}^{N-q} \alpha_{q+j,j} \prod_{k=j}^{q+j-1} \alpha_{k,k+1} \\ &= (-\lambda + \lambda^{-q} K_1^{(q)})(-\lambda)^{N-q-1} + (-1)^{N-q-1} \lambda^{N-2q-1} K_2^{(q)} \end{aligned}$$

where $K_1^{(q)} = \alpha_{q+1,1} \prod_{k=1}^{q} \alpha_{k,k+1}$ and $K_2^{(q)} = \sum_{j=2}^{N-q} \alpha_{q+j,j} \prod_{k=j}^{q+j-1} \alpha_{k,k+1}$.

Finally,

$$\begin{aligned} p_B(\lambda) &= \lambda^{N-q-1}(-1)^N [\lambda^{q+1} - K_1^{(q)} - K_2^{(q)}] \\ &= \lambda^{k_2} g(\lambda^{k_3}) \end{aligned}$$

with $k_2 = N - q - 1$, $k_3 = q + 1$ and $g(\lambda) = (-1)^N [\lambda - K_1^{(q)} - K_2^{(q)}]$. Note that $K_1^{(q)} + K_2^{(q)}$ is a simple eigenvalue of M_B.

On other hand, the matrix $M_A = (Id - M_{FF})^{-1} M_{FB}^{(q)}$ can be calculated using Eq. (3),

$$\begin{aligned} (M_A)_{ij} &= \sum_{k=1}^{N} (Id - M_{FF})^{-1}_{ik} (M_{FB}^{(q)})_{kj} \\ &= -\sum_{k=1}^{N} (-Id + M_{FF})^{-1}_{ik} \alpha_{jk} \delta_{k,j-q} \\ &= -(-Id + M_{FF})^{-1}_{i,j-q} \alpha_{j,j-q} \\ &= \alpha_{j,j-q} \prod_{k=j-q}^{i-1} \alpha_{k,k+1} \Theta(q + 1 \le j \le i + q) \end{aligned}$$

That is,

$$M_A = \left[\begin{array}{c|c} \mathbf{0} & * \\ \hline \mathbf{0} & L \end{array} \right] \qquad (5)$$

where the null blocks have q columns and the block $L \in \mathbb{R}^{N-q \times N-q}$ is defined by $L_{ij} = \alpha_{j+q,j} \prod_{k=j}^{i+q-1} \alpha_{k,k+1} \Theta(j \le i + q)$.

Using the block determinant formula, we obtain that $p_A(\lambda) = det(M_A - \lambda Id) = (-\lambda)^q det(L - \lambda Id) = (-\lambda)^q p_L(\lambda)$.

Let $\vec{x} \in \mathbb{R}^{N-q}$, then

$$\begin{aligned}(L\vec{x})_i &= \sum_{j=1}^{N-q} L_{ij} x_j \\ &= \sum_{j=1}^{N-q} x_j \alpha_{j+q,j} \prod_{k=j}^{i+q-1} \alpha_{k,k+1} \Theta(j \leq i+q) \\ &= \prod_{k=q}^{i+q-1} \alpha_{k,k+1} \sum_{j=1}^{\min(N-q,i+q)} x_j \alpha_{j+q,j} \prod_{k=j}^{q-1} \alpha_{k,k+1} \\ &= r_i \sum_{j=1}^{\min(N-q,i+q)} f_j x_j\end{aligned}$$

where $r_i = \prod_{k=q}^{i+q-1} \alpha_{k,k+1}$ and $f_j = \alpha_{j+q,j} \prod_{k=j}^{q-1} \alpha_{k,k+1}$.

Under the hypothesis that $q > \frac{N}{2} - 1$, then $\min(N-q, i+q) = N-q$, and we express

$$L\vec{x} = \langle \vec{f}, \vec{x} \rangle \vec{r}.$$

where $\vec{f} \neq 0$. Then, L has $N-q-1$ independent eigenvectors associated with the eigenvalue $\lambda = 0$ and $\vec{r}$ is an eigenvector associated with the eigenvalue $\lambda = \langle \vec{f}, \vec{r} \rangle = \sum_j \alpha_{j+q,j} \prod_{k=j}^{q+j-1} \alpha_{k,k+1} = K_1^{(q)} + K_2^{(q)}$. In conclusion, $p_A(\lambda) = \lambda^{k_1} g(\lambda)$ with $k_1 = N-1$.

Table A shows some examples of the theorem.

A.5 Faster R-CNN

Faster Region Based Convolutional Neural Network (Faster R-CNN) [3] is one of the top models used for object detection. It consists of two modules: a) Region Proposal Module (RPM) and b) Detector Module (DM). The RPM selects parts of the image which most likely contain an object (called *region proposals*). Each of these regions is then processed by the DM in which a classifier labels each region proposal and refines the bounding boxes using a regressor. Before the two modules, Faster R-CNN uses a CNN (called *backbone*) to transform the input image into a feature map with dimensions $H \times W \times Ch$ (e.g., $Ch = 512$). A schematic of the Faster R-CNN architecture is shown in Fig. A.

The goal of RPM is to learn whether an object is present in the input image at its corresponding location and estimate its size. To do this, the network uses a set of n_{anch} anchors on the input image for each location on the feature map. These anchors indicate possible objects in various sizes and aspect ratios at this location. The RPM consists of a 3×3 convolution with 512 channels (padding, stride = 1) and two independent branches. The first branch is a convolution 1×1 with $2.n_{anch}$ channels whose output ($H \times W \times n_{anch} \times 2$) is associated with the probabilities of whether a feature map point contains an object for each of the anchors (*confidence scores*). The other branch is a convolution 1×1 with $4.n_{anch}$ channels whose output ($H \times W \times n_{anch} \times 4$) corresponds to the 4 regression coefficients of each of the anchors (x, y coordinates of the center, height, width) for every point on the feature map. These regression coefficients are used to improve the prediction of the position and size of the anchors that contain objects.

At this point, the RPM returns a list of $H.W.n_{anch}$ of boxes with its corresponding confidence score and regression coefficients. From this list, only those boxes that match

Layers N	Distance q	Biological	Artificial	Order of g	Example
2	0	$g(\lambda)$	$g(\lambda)$	2	
	1	$g(\lambda^2)$	$\lambda g(\lambda)$	1	
3	0	$g(\lambda)$	$g(\lambda)$	3	
	1	$\lambda g(\lambda^2)$	$\lambda^2 g(\lambda)$	1	
	2	$g(\lambda^3)$	$\lambda^2 g(\lambda)$	1	
4	0	$g(\lambda)$	$g(\lambda)$	4	
	1	$g(\lambda^2)$	$\lambda^2 g(\lambda)$	2	
	2	$\lambda g(\lambda^3)$	$\lambda^3 g(\lambda)$	1	
	3	$g(\lambda^4)$	$\lambda^3 g(\lambda)$	1	
5	0	$g(\lambda)$	$g(\lambda)$	5	
	1	$\lambda g(\lambda^2)$	$\lambda^3 g(\lambda)$	2	
	2	$\lambda^2 g(\lambda^3)$	$\lambda^4 g(\lambda)$	1	
	3	$\lambda g(\lambda^4)$	$\lambda^4 g(\lambda)$	1	
	4	$g(\lambda^5)$	$\lambda^4 g(\lambda)$	1	
6	0	$g(\lambda)$	$g(\lambda)$	6	
	1	$g(\lambda^2)$	$\lambda^3 g(\lambda)$	3	
	2	$g(\lambda^3)$	$\lambda^4 g(\lambda)$	2	
	3	$\lambda^2 g(\lambda^4)$	$\lambda^5 g(\lambda)$	1	
	4	$\lambda g(\lambda^5)$	$\lambda^5 g(\lambda)$	1	
	5	$g(\lambda^6)$	$\lambda^5 g(\lambda)$	1	

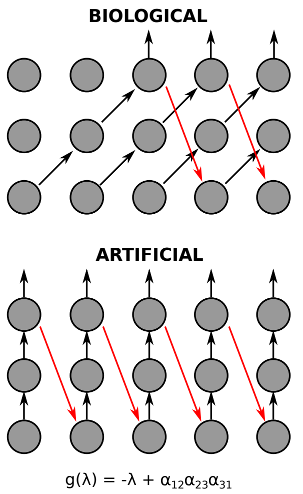

Table A. Comparison between the characteristic polynomials for networks with N layers and only feedback connections of distance q. For all N, in the case $q = 0$, it is obtained that $g(\lambda) = \prod_{i=1}^{N}(\lambda - \alpha_{ii})$. Note that in all cases the Eq. (9) is verified. The coefficients of g depend on the entries of the matrices involved. However, the order of g only depends on N and q. In the example, we show the scheme for the biological and artificial implementation for a network with $N = 3$ layers and feedback connections of distance $q = 2$ (see highlighted row). For this case, the polynomial g is linear (order 1).

specific selection criteria are used by the DM. For example, boxes with height/width smaller than a threshold, or boxes that cross the boundary are ignored. Another way to filter the boxes is to compare the confidence score with a threshold. The high confidence score is associated with anchor boxes that probably contain an object; while low score indicates that the anchor box contains no object (background). However, if the score is somewhere in between, the anchor box could contain a partial object and is not a good reference to locate the object; then, it is deleted. One last option is to use Non-Max Suppression (NMS), which identifies boxes with high IoU and removes the anchor box with lower objectness score. Usually, filtering gives about $\sim 2k$ proposals per image.

The Detector Module consists of an ROI pooling layer and fully connected layers followed by two branches for classification and bounding box regression. It uses a certain number of N_{prop} of proposals from the RPN depending on whether it is in the training or evaluation stage. For the training stage, all proposals are used; while for the validation and test stage, only the top $N_{prop} \sim 10,100$ proposals are selected. For each proposal, an ROI pooling layer takes the region corresponding $(h_r \times w_r)$ from the feature map. Then, it divides this region into a fixed number of sub-windows (e.g. 49 windows $= 7 \times 7$) and applies max-pooling over each sub-window. The output of the ROI pooling layer has a fixed size $(N_{prop} \times 7 \times 7 \times Ch)$, which flattens out $(N_{prop} \times 25088)$. Finally, this result goes through two fully connected layers and is fed into the classification and regression branches. The classification layer has C units (one per class in the detection task); while the bounding box regressor consists of $4.C$ units

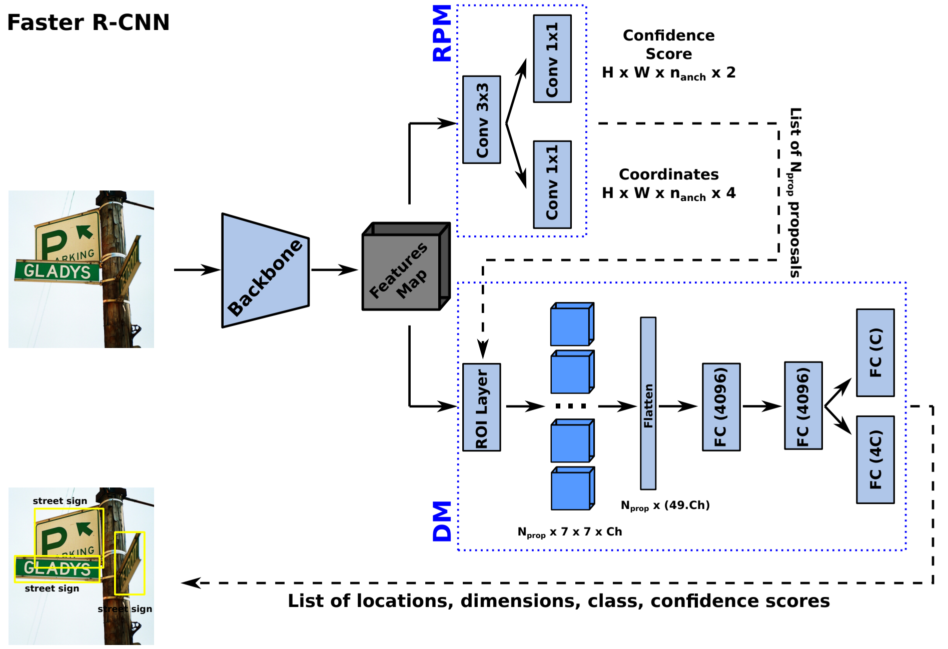

Fig A. Faster R-CNN architecture. The model consists of a backbone (CNN) and two main modules: a) Region Proposal Module (RPM) and Detector Module (DM). The goal of the RPM is to detect the objects; while the DM takes care of classifying them. Details about the components are presented in the main text. Abbreviations: FC (m) - Full connected layers with m units.

to refine the location and dimensions of each bounding box and class specific. An additional step is to use another NMS in which anchor boxes with both the same class and high IoU are compared, and boxes with lower confidence scores are eliminated.

The final output of the Faster R-CNN is a list of bounding boxes with their locations, dimensions, class labels, and confidence scores. It is jointly trained with four losses: 1) RPM classification, 2) RPM regression, 3) DM classification, and 4) DM regression. For more information about the network, it is recommended to read [3].

A.6 ResNet stages

ResNet architecture is a type of feedforward neural network consisting of a set of building blocks [4]. Each block is made up of 2 or 3 convolutions and residual connections. A set of blocks that operate sequentially is called the 'ResNet stage'. Table B shows the architecture of ResNet-50 and ResNet-18 that we use for our recurrent CNNs. In the Eq. (6), F_L represents a 'ResNet stage'. For example, F_1 for ResNet-50 is the combined operation of applying a 7×7 convolution (64 filters) and 3×3 max-pooling.

A.7 Metrics

Intersection over Union (IoU) is a value that quantifies the degree of overlap between two boxes A and B

$$\mathrm{IoU}(A, B) = \frac{\mathrm{area}(A \cap B)}{\mathrm{area}(A \cup B)}. \tag{6}$$

Stage	ResNet-50	ResNet-18
1	7×7, 64	3×3, 64
	3×3 max-pooling	
2	$\begin{bmatrix} 1 \times 1, 64 \\ 3 \times 3, 64 \\ 1 \times 1, 256 \end{bmatrix} \times 3$	$\begin{bmatrix} 3 \times 3, 64 \\ 3 \times 3, 64 \end{bmatrix} \times 2$
3	$\begin{bmatrix} 1 \times 1, 128 \\ 3 \times 3, 128 \\ 1 \times 1, 512 \end{bmatrix} \times 4$	$\begin{bmatrix} 3 \times 3, 128 \\ 3 \times 3, 128 \end{bmatrix} \times 2$
4	$\begin{bmatrix} 1 \times 1, 256 \\ 3 \times 3, 256 \\ 1 \times 1, 1024 \end{bmatrix} \times 6$	$\begin{bmatrix} 3 \times 3, 256 \\ 3 \times 3, 256 \end{bmatrix} \times 2$
5	$\begin{bmatrix} 1 \times 1, 512 \\ 3 \times 3, 512 \\ 1 \times 1, 2048 \end{bmatrix} \times 3$	$\begin{bmatrix} 3 \times 3, 512 \\ 3 \times 3, 512 \end{bmatrix} \times 2$

Table B. Architectures of ResNet. Building blocks are shown in brackets with the number of blocks stacked for each stage. For more details, see [4].

IoU is mainly used in applications related to object detection. For example, it is used in different stages of Faster R-CNN (see Section A.5). In the validation stage, Faster R-CNN generates a list of N boxes with the corresponding location, dimensions (x, y, h, w), class c, and confidence score. When the confidence score associated with a box is greater than a threshold score s, we say that *the model predicted a bounding box pd*. Consider the subset of bounding boxes pd predicted by the model with class c and an IoU-threshold $\mu \in [0, 1]$; then, True Positive (TP) and False Positive (FP) cases are defined by:

- TP: If the class c matches the class of a ground truth gt and $\mu \leq \mathrm{IoU}(pd, gt)$ - see Fig. Bc,e*.
- FP: There are three scenarios. First, the class c matches the class of a ground truth gt but $\mathrm{IoU}(pd, gt) < \mu$ - see Fig. Bb,f. The other case is when there already is a bounding box with higher IoU for the ground truth - see Fig. Be. The last case is when c does not match the class of gt - see Fig. Bd (red).

On the other hand, a False Negative (FN) case is defined when the model did not predict a bounding box at a certain position and it was wrong (i.e. a ground truth bounding box existed at that position) - see Fig. Ba,b,d. Note that these definitions depend strongly on the thresholds s and μ, and the class c.

With the above values, the precision and recall can be calculated for each value of s, μ, c:

$$p(s, \mu, c) = \frac{TP}{TP + FP}, \qquad r(s, \mu, c) = \frac{TP}{TP + FN}. \tag{7}$$

The area under the precision-recall curve $p_{\mu,c} = p_{\mu,c}(r_{\mu,c})$ is $\mathrm{AP}_{\mu,c} = \int_0^1 p_{\mu,c}(r)dr$. Finally, Mean Average Precision (mAP_μ) is the average of $\mathrm{AP}_{\mu,c}$ of each class c. The mAP incorporates the trade-off between precision and recall and considers both false positives (FP) and false negatives (FN). This property makes mAP a suitable metric for most detection applications.

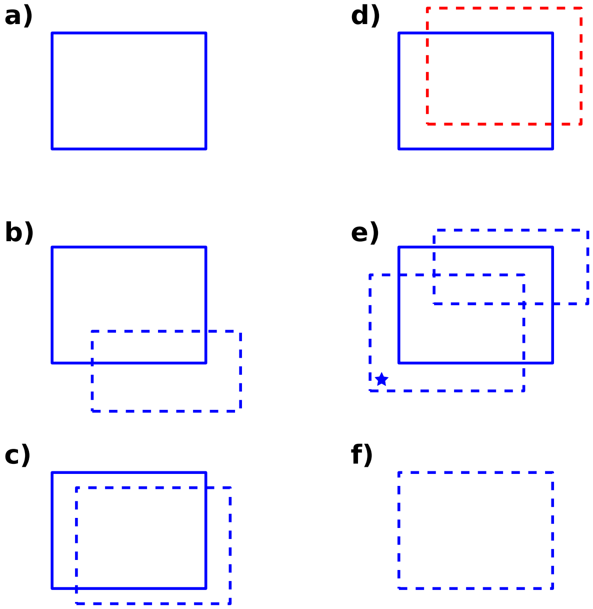

Fig B. Scenarios in object detection. The boxes with solid lines and dashed lines indicate the ground truth and the predictions, respectively. Blue and red colors indicate different classes (e.g., dogs and cats). In the main text, it is indicated if the predictions correspond to TP, FP, or FN. Note that in (b) the prediction is a FP case and there is a FN case, simultaneously. On the other hand, in (d), the prediction is a FP for the red class but there is a FN case for the class blue.

On the other hand, the area under the recall-IoU curve is $\mathrm{AR}_{s,c} = \int_{0.5}^{1} r(s, \mu, c) d\mu$ and Mean Average Recall (mAR_s) is defined as the mean of $\mathrm{AR}_{s,c}$ across all classes. In Section 3.7, we show the performance of the CNNs in terms of mAP and mAR. For these metrics, all confidence intervals (95%) were estimated utilizing a bootstrap procedure, resulting in a relative standard error of less than 2%.

Both mAR and mAP can be calculated on the set of all detected objects or on a subset of them. For example, we can separate objects based on their size. A 'small object' covers an area of less than 32^2 pixels; 'medium objects' cover an area of 32^2 to 96^2; and 'large objects' cover an area greater than 96^2.

References

1. Palani S. The z-Transform Analysis of Discrete Time Signals and Systems. In: Signals and Systems. Springer, Cham 2023.

2. Arfken GB, Weber HJ. Mathematical Methods for Physicists, 6th Edition. Amsterdam, Heidelberg: Academic Press; 2005.

3. Shaoqing Ren, Kaiming He, Ross Girshick, and Jian Sun. Ren S, He K, Girshick R, Sun J. Faster R-CNN: Towards Real-Time Object Detection with Region Proposal Networks. arXiv; 2016.

4. He K, Zhang X, Ren S, Sun J. Deep Residual Learning for Image Recognition. arXiv; 2015.
